# Acyl radical to rhodacycle addition and cyclization relay to access butterfly flavylium fluorophores

**DOI:** 10.1038/s41467-019-13611-6

**Published:** 2019-12-11

**Authors:** Jiangliang Yin, Yuming Zhang, Jian Li, Lei Zhu, Yu Lan, Jingsong You

**Affiliations:** 1 Key Laboratory of Green Chemistry and Technology of Ministry of Education, College of Chemistry, Sichuan University, 29 Wangjiang Road, 610064 Chengdu, PR China; 2 School of Chemistry and Chemical Engineering, Chongqing University, 400030 Chongqing, PR China

**Keywords:** Organic chemistry, Reaction mechanisms, Synthetic chemistry methodology

## Abstract

Transition metal-catalyzed C–H activation and radical reactions are two versatile strategies to construct diverse organic skeletons. Here we show the construction of a class of flavylium fluorophores via the merge of radical chemistry and C–H activation starting from (hetero)aryl ketones and alkynes. This protocol is not only applicable to aryl ketones but also to heteroaryl ketones such as thiophene, benzothiophene and benzofuran, thus leading to structural diversity. Mechanism studies, including control experiments, intermediate separation, radical trapping, EPR and ESI-HRMS experiments, demonstrate that the key step lies in the addition of the acyl radical generated by the copper-catalyzed C–C bond cleavage of aryl ketone to the rhodacycle formed via the C–H activation of aryl ketone. The flavylium fluorophores feature butterfly symmetrical configuration, nearly planar skeleton and delocalized positive charge, and exhibit intriguing photophysical properties, such as tunable absorption and emission wavelengths and high quantum yields.

## Abstract (web summary)

Structural diversity of organic fluorophores is of importance for several applications (fluorescent markers, photosensitizers, etc.). Here the authors report a method to merge radical chemistry with C–H activation to construct a brand-new class of flavylium fluorophores starting from (hetero)aryl ketones and alkynes.

## Introduction

**Fig. 1:**
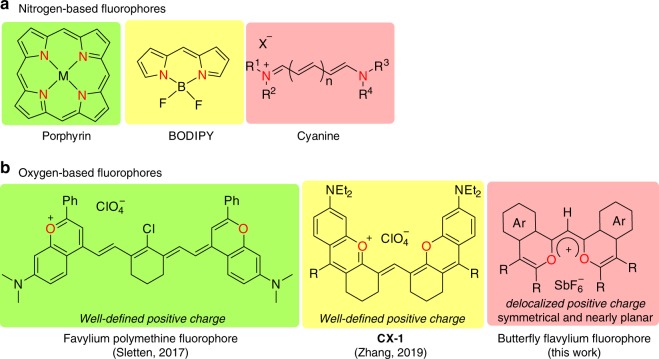
Representative organic fluorophores. **a** Nitrogen-based fluorophores. **b** Oxygen-based fluorophores.

Due to attractive electronic, optical, electrochemical and magnetic properties, organic fluorophores have been considered as promising candidates for applications, such as fluorescent markers, photosensitizers, nonlinear optical materials and nano-electronic devices^1–13^. Therefore, diverse kinds of organic fluorophores have been discovered and developed, among which nitrogen-based porphyrins^1,2^, BODIPYs^3–5^ and cyanine derivatives^6,7^ exhibit extensive and outstanding properties (Fig. 1a). In recent years, oxygen-doped fluorophores based on pyrylium framework begin to attract attention because of tunable emission wavelength, high quantum yield and water solubility, making them as ideal optical-imaging reagents (Fig. 1b)^8–13^. From both the fundamental and application viewpoints, a study of the structural diversity of organic fluorophores is doubtlessly of great importance.

Transition metal-catalyzed C–H activation and radical reactions have been developed as two versatile tools for the construction of organic functional skeletons^14–35^. The metal-catalyzed C–H activation reactions proceed via the formation of carbon–metal bond, typically classified as charged species—involving ionic reactions, while the key step of the radical reactions involves an electrophilic addition of a radical species to arene, alkene, or alkyne. Undoubtedly, the combination of ionic and radical reactions would create new chemical transformations and thereby produce complex molecules with high reaction efficiency. Despite an appealing strategy, it is challenging to achieve a well-organized reaction relay because the presence of the highly reactive radical species may give rise to an uncontrollable, complicated reaction process. Given the weak coordinating ability and keto–enol tautomerism, as well as easily being oxidized to radical species, aryl ketones are considered as an ideal element to implement diverse chemical transformations^20–23,32,33^. By using aryl ketones and alkynes as substrates, Glorius and Cheng independently reported the rhodium-catalyzed C2–H activation/annulation reactions to deliver indenol and fulvene derivatives^20,21^. In our previous work, carbohelicenes were synthesized by a C–H activation/radical approach/C–H activation relay of α-acetylnaphthalenes with alkynes^35^.

**Fig. 2:**
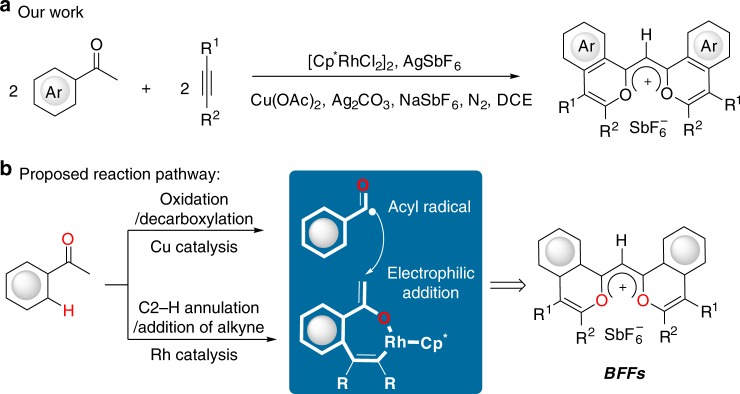
Merging radical chemistry with C–H activation to construct BFFs. **a** In this report, one step synthesis of BFFs. **b** The proposed reaction pathway.

Herein, we describe the addition of an acyl radical generated by the copper-catalyzed sequential oxidation/C–C bond cleavage of (hetero)arylketones to the rhodacycle to produce butterfly flavylium fluorophores (BFFs) with symmetrical and nearly planar skeleton with delocalized positive charge (Fig. 2).

## Results

### Analysis of structure and DFT calculation

**Fig. 3:**
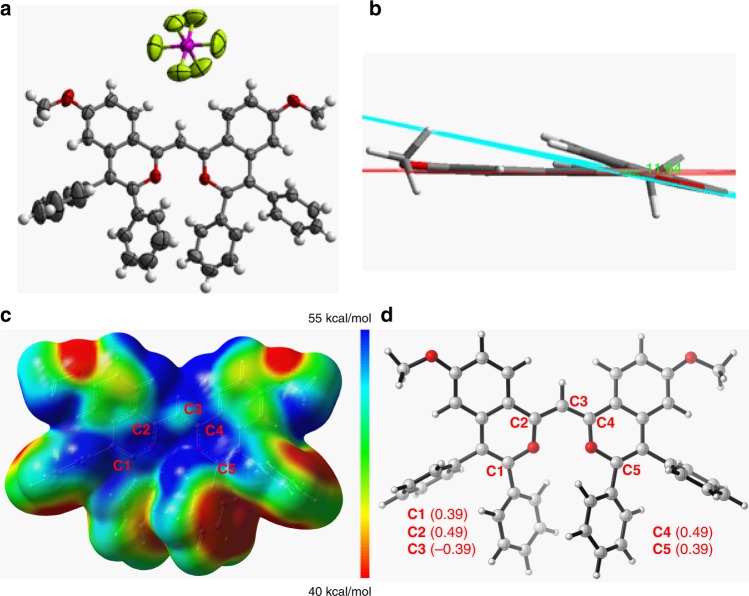
X-ray single crystal diffraction analysis and DFT calculation of 3aa. **a** Top view of X-ray single crystal structure of **3aa**. **b** Side view of X-ray single crystal structure of **3aa**. **c** Electrostatic potential maps of **3aa**. **d** Calculated NBO atomic charge distribution of cationic **3aa**.

In the investigation of the reaction of 4-methoxyacetophenone with diphenylacetylene, the BFF **3aa** was unexpectedly isolated in 8% yield by using 5 mol% of [Cp*RhCl_2_]_2_ as the catalyst, Ag_2_O as the oxidant, Cu(OAc)_2_·H_2_O as the additive, and DCE as the solvent in the presence of 20 mol% of AgSbF_6_ at 150 °C (Supplementary Table 1, entry 1). X-ray single crystal analysis of **3aa** shows a nearly planar skeleton with a little angle of 11.6° between the two pyrylium rings (Fig. 3a, b). The length of C2–C3 bond (1.389(5) Å) is close to that of C3–C4 bond (1.372(5) Å), which are between the lengths of C–C single bond (1.455 Å) and C = C double bond (1.326 Å)^36^. The ^1^H NMR spectrum of **3aa** shows a symmetrical configuration, suggesting that the positive charge is delocalized equally around the skeleton. In addition, density functional theory (DFT) calculation was employed to evaluate the intrinsic characteristic of cationic **3aa** (Supplementary Data 1)^37,38^. As shown in Fig. 3c, d, the natural population analysis and natural bond orbital (NBO) atomic charge distribution further illustrate that the positive charge of **3aa** is mostly delocalized by the two pyrylium rings.

### Optimization of the reaction conditions

With the butterfly flavylium scaffold in hand, optimization of the reaction conditions was next conducted (Supplementary Table 1). Considering the zwitterionic form of the product, 1.5 equivalents of NaSbF_6_ were added into the reaction system, improving the yield of **3aa** to 45% (Supplementary Table 1, entry 3). The copper salt is important for this reaction and no desired product could be detected without the addition of copper salt (Supplementary Table 1, entry 4). Subsequently, various copper species, such as CuBr_2_, CuO, CuCl, Cu(OAc), and Cu_2_O were screened (Supplementary Table 1, entries 5–9). **3aa** was obtained in 49% yield when 0.04 mmol of Cu(OAc)_2_·H_2_O was used (Supplementary Table 1, entry 10). Oxidant Ag_2_CO_3_ could further improve the yield of **3aa** to 63% (Supplementary Table 1, entry 15). Solvents are critical for this reaction and other solvents, such as toluene, tetrahydrofuran, 1, 4-dioxane, and *N*,*N*-dimethylformamide did not deliver the product (Supplementary Table 1, entries 17–20). The yield of **3aa** could reach 73% by decreasing the temperature from 150 to 100 °C (Supplementary Table 1, entry 24).

### Substrate scope

**Fig. 4:**
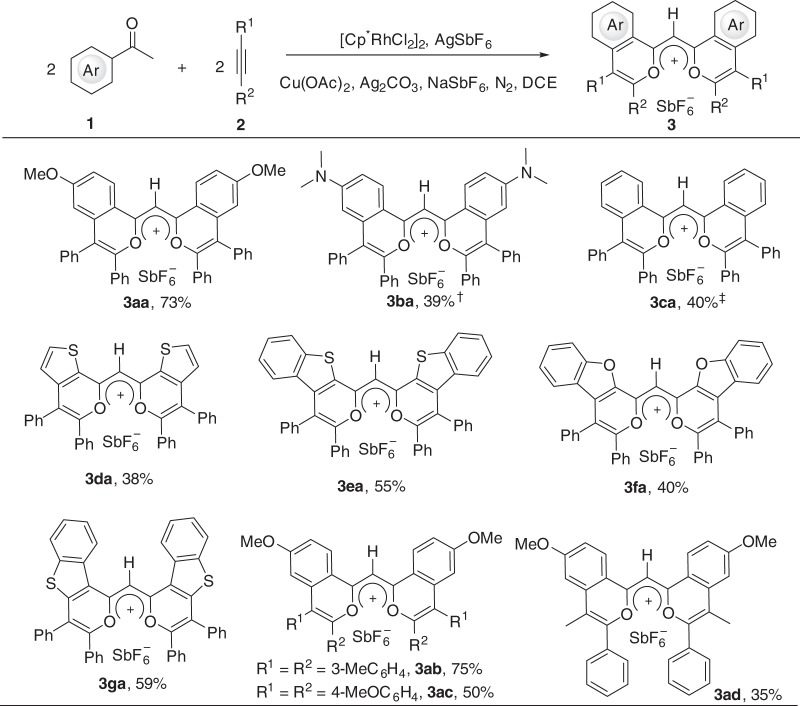
Substrate scope. Reaction conditions: **1** (0.2 mmol), **2** (0.3 mmol), [Cp*RhCl_2_]_2_ (5 mol%), AgSbF_6_ (20 mol%), Ag_2_CO_3_ (0.3 mmol), Cu(OAc)_2_·H_2_O (0.04 mmol), NaSbF_6_ (0.15 mmol), and DCE (0.5 mL) at 100 °C for 12 h under N_2._^†^At 75 °C for 4 h. ^‡^At 150 °C.

To assemble a structurally diverse library of the BFFs, the substrate scope was conducted. As shown in Fig. 4, the cascade annulation of (hetero)aryl ketones with alkynes allowed a relatively broad substrate scope, producing a diverse family of BFFs in moderate to good yields. The aryl ketones with an electron-donating group at the *para* position such as *N,N*-dimethyl could deliver the desired fluorophore (**3ba**) in 39% yield. 3-Methylacetophenone and 3,4-dimethoxyacetophenone could give the corresponding fluorophores, but the pure products were not obtained because of their instability during column chromatography. Delightedly, the heteroaryl ketones such as 2-acetylthiophene, 2-acetylbenzo[*b*]thiophene, 2-acetylbenzo[*b*]furan, and 3-acetylbenzo[*b*]thiophene could afford the corresponding fluorophores (**3da**-**3ga**) in moderate yields. The good compatibility of heteroaryl ketones enriches the structural diversity. Next, the scope of the alkyne derivatives was investigated. The electron-donating group such as methyl and methoxy could be tolerated, producing the desired products (**3ab**-**3ac**) in moderate to good yields. The unsymmetrical alkyl aryl alkyne delivered **3ad** with a good regioselectivity and other isomers were not detected.

### Mechanism of investigation

**Fig. 5:**
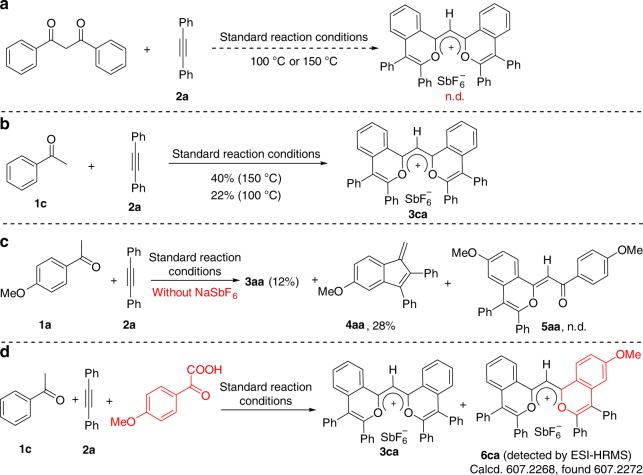
Control experiments for the reaction mechanism. **a** The reaction of 1,3-diphenylpropane-1,3-dione with **2a** was performed under standard reaction conditions. **b** The reaction of **1c** with **2a** was performed under the standard reaction conditions at 100 or 150 °C. **c** The reaction of **1a** with **2a** was performed without the addition of the extra anion source NaSbF_6_. **d** The reaction of **1c**, **2a**, and 4-methoxybenzoyl formic acid was performed under the standard reaction conditions.

To get a clearer perception of this cascade process, a set of control experiments were performed (Fig. 5). Firstly, under the standard reaction conditions, acetophenone, respectively, delivered the BFF **3ca** in 22% yield at 100 °C and 40% yield at 150 °C, while 1,3-diphenylpropane-1,3-dione did not deliver the corresponding product either at 100 or 150 °C (Fig. 5a, b). These results indicated that 1,3-dicarbonyl compound was not the intermediate of this reaction. Secondly, under otherwise identical reaction conditions, the absence of counteranion source NaSbF_6_ led to **3aa** in 12% yield and the fulvene derivative **4aa** in 28% yield (Fig. 5c). Under the standard reaction conditions, the reaction of **1a** with **2a** could deliver a trace amount of the fulvene derivative **4aa**. The formation of **4aa** demonstrated that the C2–H activation process of arylketone existed in this reaction system. The ESI-HRMS experiment did not detect **5aa**, implying that the cascade reaction might be a successive process rather than a step by step process (Fig. 5c). In a step by step process, a stable reaction intermediate could be isolated through condition control and the final product could be obtained in an acceptable yield by using the reaction intermediate as the starting material. Thirdly, under the standard reaction conditions, the reaction of **1a**, **2a**, and 4-methoxybenzoyl formic acid gave a mixture of **3ca** and the unsymmetric product **6ca**, which was detected by ESI-HRMS (Fig. 5d and Supplementary Fig. 3). This result suggested that the benzoylformic acid could be one of the reaction intermediates and the deprivation of the carbon atom could be related to the decarboxylation process.

**Fig. 6:**
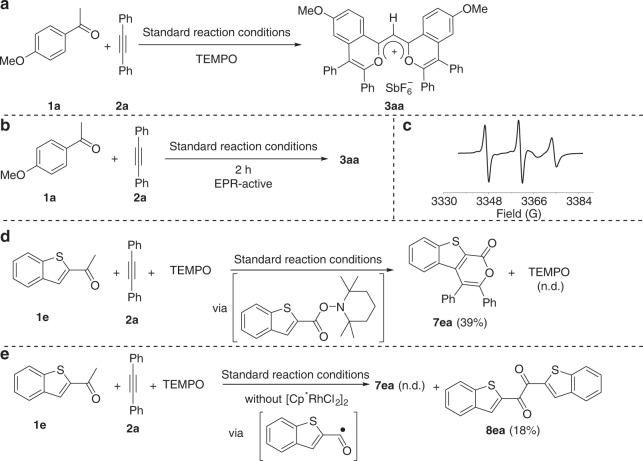
Detection of radical intermediates. **a** Radical trapping experiments. **b** EPR experiments of the reaction **1a** with **2a**. **c** EPR spectrum of the reaction mixture of **1a** and **2a** under standard reaction conditions. **d** The reaction of **1e**, **2a**, and TEMPO under the standard reaction conditions. **e** The reaction of **1e**, **2a**, and TEMPO without the addition of catalyst [Cp*RhCl_2_]_2_.

Based on the above results, we infer that this transformation might involve two main processes: (a) the generation of rhodacycle by the C2–H activation of arylketone; and (b) the generation of acyl radical by the oxidation/decarboxylation sequence of arylketone. To gain more insights into the radical process, radical trapping experiments were carried out. The yield of **3aa** decreased from 52% to trace with increasing amount of radical quencher (2,2,6,6-tetramethylpiperidin-1-yl)oxyl (TEMPO), suggesting the possible radical pathway (Fig. 6a and Supplementary Table 2). Subsequently, EPR analysis indicated that the reaction of **1a** with **2a** was EPR-active, while no signals were observed in the absence of both **1a** and **2a** (Fig. 6b, c and Supplementary Fig. 6a, b). Under the standard reaction conditions, the product **3aa** had only a weak EPR signal (Supplementary Fig. 6c). Furthermore, the reaction of **1e**, **2a** and TEMPO could deliver the isocoumarin product **7ea** in 39% yield, while only a trace amount of **7ea** could be detected without the addition of TEMPO (Fig. 6d). This result indicated that the adduct of acyl radical with TEMPO could be the reaction intermediate for the generation of **7ea** (for details, see Supplementary Fig. 4). In addition, without the addition of catalyst [Cp*RhCl_2_]_2_, the reaction of **1e**, **2a** and TEMPO could produce **8ea** in 18% yield, demonstrating the generation of acyl radical (Fig. 6e and Supplementary Fig. 5). These results clearly demonstrated that the acyl radical species is involved in the cascade reaction.

**Fig. 7:**
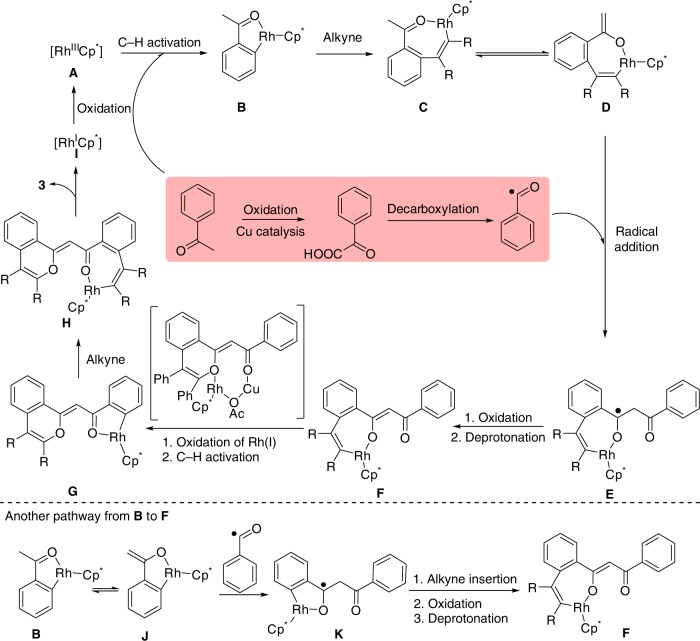
Plausible catalytic cycle. The possible mechanism involves the Rh-catalyzed C2–H activation of aryl ketone to generate the rhadocycle and the Cu-catalyzed oxidation/C–C bond cleavage of aryl ketone to produce the acyl radical.

Based on the experimental observations and the reported literature, the reaction pathway is proposed in Fig. 7. Firstly, the reactive [Rh^III^Cp^*^] **A** is formed via the reaction of [Cp*RhCl_2_]_2_ with AgSbF_6_, followed by the cyclometalation and alkyne insertion. The resulting intermediate **C** could undergo a keto–enol tautomerism to generate the intermediate **D**, which is more easily to be trapped by a radical species. At the same time, the (hetero)aryl ketone is oxidized to (hetero)aryl glyoxylic acid, followed by the generation of acyl radical through a decarboxylation under copper catalysis^39,40^. The selective electrophilic addition of the acyl radical to the rhodacycle **D** gives the radical species **E**, followed by the sequential oxidation and deprotonation process to form intermediate **F**. In the synthesis of indenol and fulvene derivatives reported by Glorius, the catalytic Cu(OAc)_2_·H_2_O plays the role in the release of the rhodium catalyst in a transmetalation event^20^. We propose that one of the key roles of Cu(OAc)_2_·H_2_O is to promote the intramolecular rhodium migration, accompanied by the oxidation of Rh(I) to Rh(III) and the C–H activation of another (hetero)arylketone to form intermediate **G**. The second alkyne inserts into **G** to produce **H**, followed by a reductive elimination to yield the butterfly flavylium fluorophore. By the oxidation of Ag_2_CO_3_, the active Rh (III) species is regenerated. There is another possible pathway from intermediate **B** to **F** as shown in Fig. 7. The acyl radical may attack the intermediate **J** generated by a keto–enol tautomerism of **B** prior to the first alkyne insertion.

### Photophysical properties of BFFs

**Fig. 8:**
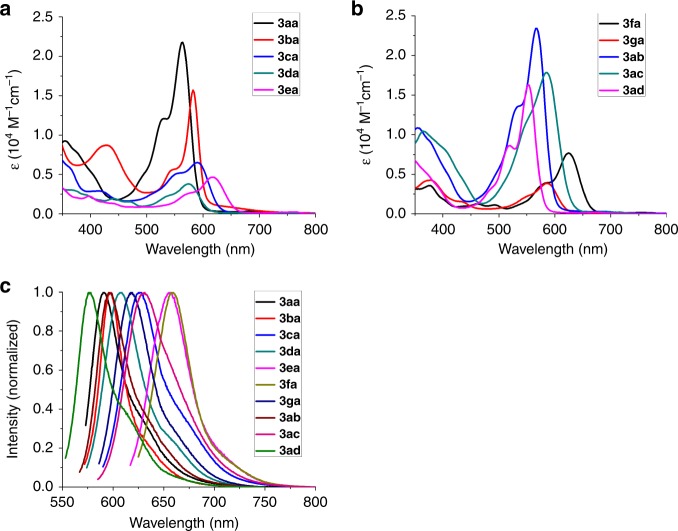
Absorption and emission spectra of the BFFs in CH_2_Cl_2_ at 40.0 μM. **a** The absorption spectra of **3aa**, **3ba**, **3ca**, **3da**, and **3ea**. **b** The absorption spectra of **3fa**, **3ga**, **3ab**, **3ac** and **3ad**. **c** The emission spectra of all BFFs.

With the diverse BFFs in hand, the photophysical properties of fluorophores, including UV–Vis absorption, fluorescence emission, extinction coefficients, excitation, and quantum yield, were measured in CH_2_Cl_2_ (Supplementary Table 3 and Supplementary Fig. 7). As shown in Fig. 8 and Supplementary Table 3, the BFFs show tunable absorption and emission wavelengths and high quantum yields in CH_2_Cl_2_. The quantum yields of the fluorophores **3ca**, **3ea**, **3fa**, and **3ac** could reach 46%, 59%, 63%, and 49% even with emission maxima of 626, 656, 660, and 631 nm, respectively.

## Discussion

In summary, a class of BFFs has been developed through the merging of C–H activation and radical chemistry. Because both (hetero)arylketones and alkynes are easily available and structurally diverse, the structures of the BFFs are easily subjected to chemical modification. These fluorophores exhibit intriguing photophysical properties, such as tunable absorption and emission wavelengths, high quantum yield and zwitterionic form, rendering them potential applications as luminescence materials. The rapid gateway to BFFs described herein has further exemplified the power of the combination of C–H activation and radical chemistry in the exploration of organic functional materials.

## Methods

### General procedure for the synthesis of BFFs

A Schlenk tube with a magnetic stir bar was charged with [Cp*RhCl_2_]_2_ (0.005 mmol), AgSbF_6_ (0.02 mmol), Ag_2_CO_3_ (0.3 mmol), Cu(OAc)_2_·H_2_O (0.04 mmol), NaSbF_6_ (0.15 mmol), arylketone (0.2 mmol), alkyne (0.3 mmol), and DCE (0.5 mL) under an N_2_ atmosphere. The resulting solution was stirred at room temperature for 10 min and then at the indicated temperature for the indicated time. The resulting solution was cooled to ambient temperature, diluted with 10 mL of dichloromethane. The obtained organic extracts was evaporated under reduced pressure and the residue was absorbed into small amounts of silica gel. Purification was performed by column chromatography on silica gel to provide product **3**. The reaction vessel is under pressure and could possibly explode because of higher reaction temperature than the boiling point of DCE.

## Supplementary information

Supplementary Information

Description of Additional Supplementary Files

Supplementary Data 1

## Data Availability

Experimental procedures and characterization data are available within this article and Supplementary Information. The X-ray crystallographic coordinates for structures reported in this article have been deposited at the Cambridge Crystallographic Data Centre (CCDC) under deposition numbers CCDC 1914708 (**3aa**) and CCDC 1917431 (**3ad**). These data can be obtained free of charge from The Cambridge Crystallographic Data Centre via www.ccdc.cam.ac.uk/data_request/cif.
