# Supplementary material for: Acyl radical to rhodacycle addition and cyclization relay to access butterfly flavylium fluorophores
Source: Nat Commun. 2019 Dec 11;10:5664. doi: 10.1038/s41467-019-13611-6 (PMC6906420; doi:10.1038/s41467-019-13611-6)
Supplement: Supplementary file 1 — Supplementary Information [file 41467_2019_13611_MOESM1_ESM.pdf]

Supplementary Information

Acyl radical to rhodacycle addition and cyclization relay to access butterfly flavylium fluorophores

Yin et al

Supplementary Figures

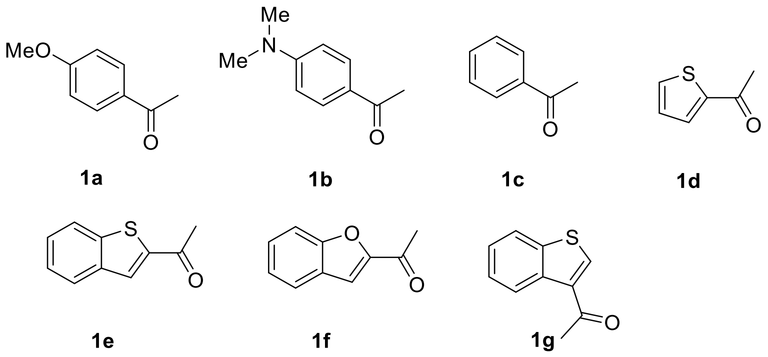

Supplementary Figure 1. Structures of (hetero)aryl ketones

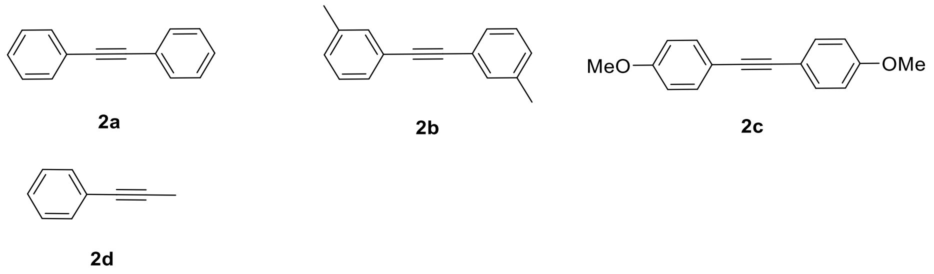

Supplementary Figure 2. Structures of alkynes

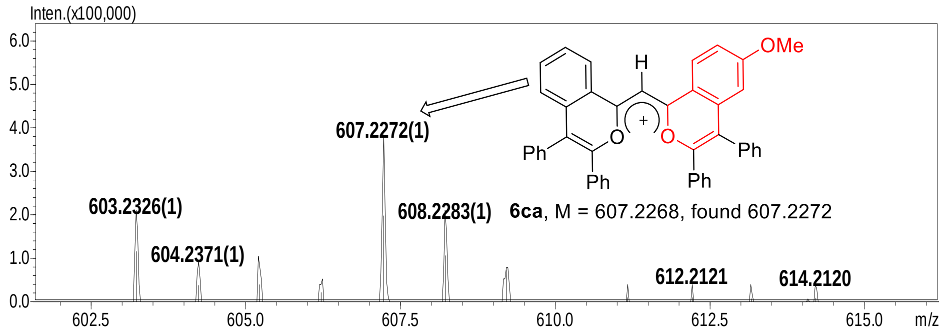

Supplementary Figure 3. ESI-HRMS spectrum for the detection of 6ca.

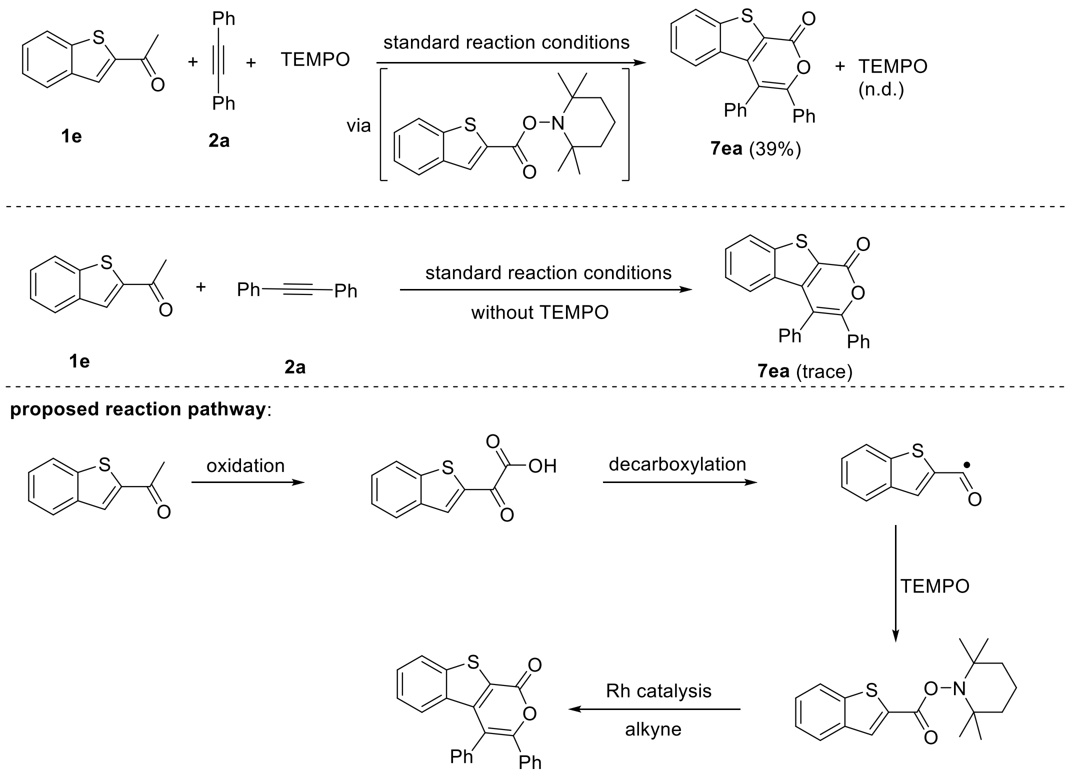

Supplementary Figure 4. Proposed reaction pathway for the formation of 7ea.

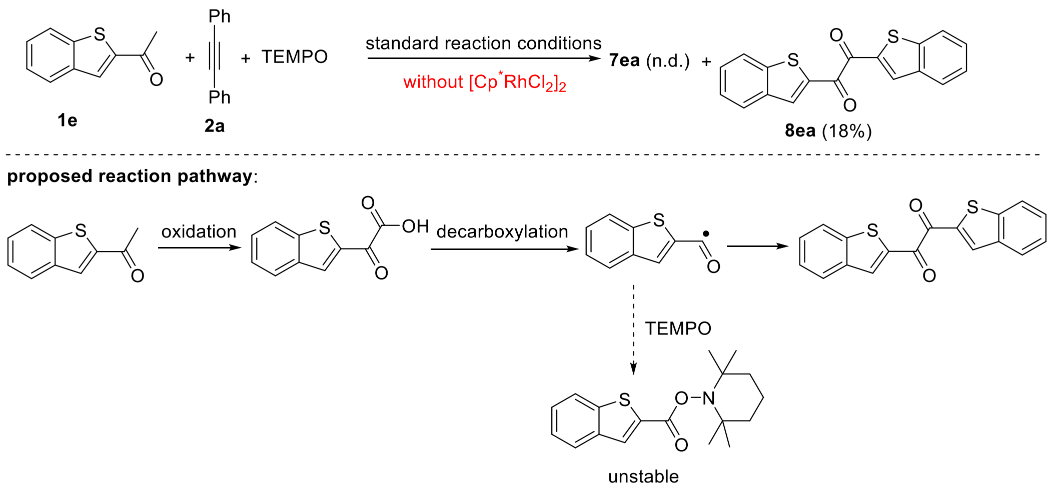

Supplementary Figure 5. Proposed reaction pathway for the formation of 8ea.

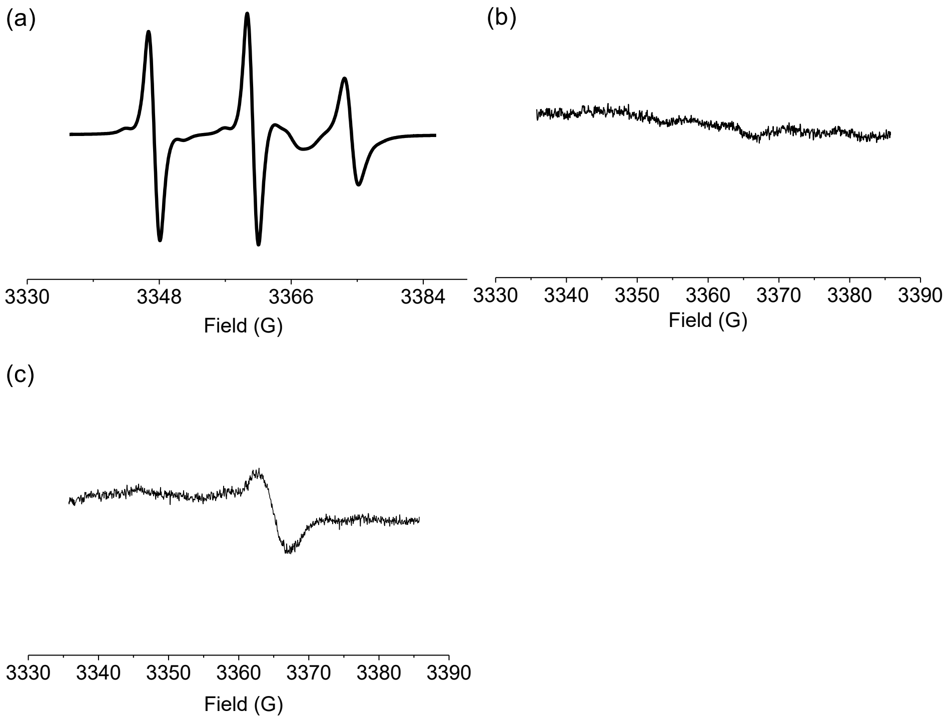

Supplementary Figure 6. EPR spectra for the radical detection. a, EPR spectrum for the reaction mixture of **1a** with **2a** under standard reaction conditions. **b,** EPR spectrum for the reaction mixture without the addition of **1a** and **2a** under standard reaction conditions. **c,** EPR spectrum for the reaction mixture of **3aa** under standard reaction conditions.

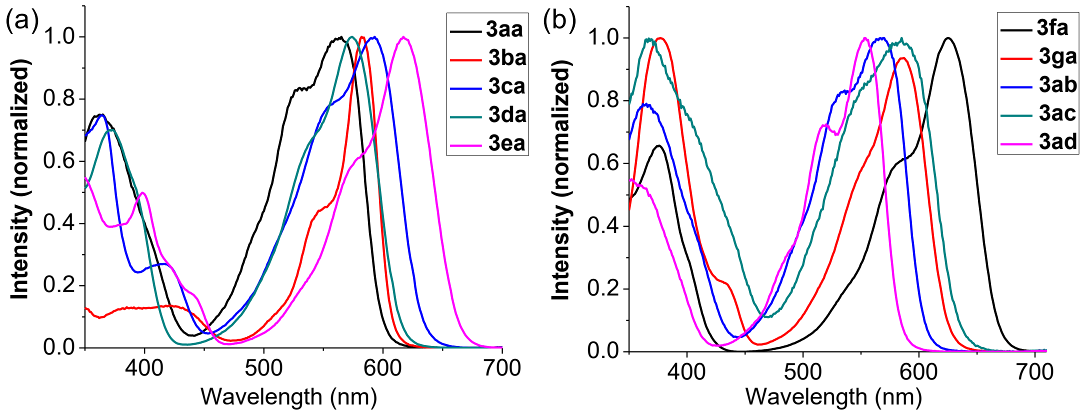

Supplementary Figure 7. Excitation spectra of the BFFs in CH_2Cl_2 at 40.0 μM. a, The excitation spectra of **3aa**, **3ba**, **3ca**, **3da** and **3ea**. **b,** The excitation spectra of **3fa**, **3ga**, **3ab**, **3ac** and **3ad**. Excitation spectra were detected upon the maximum emission wavelength.

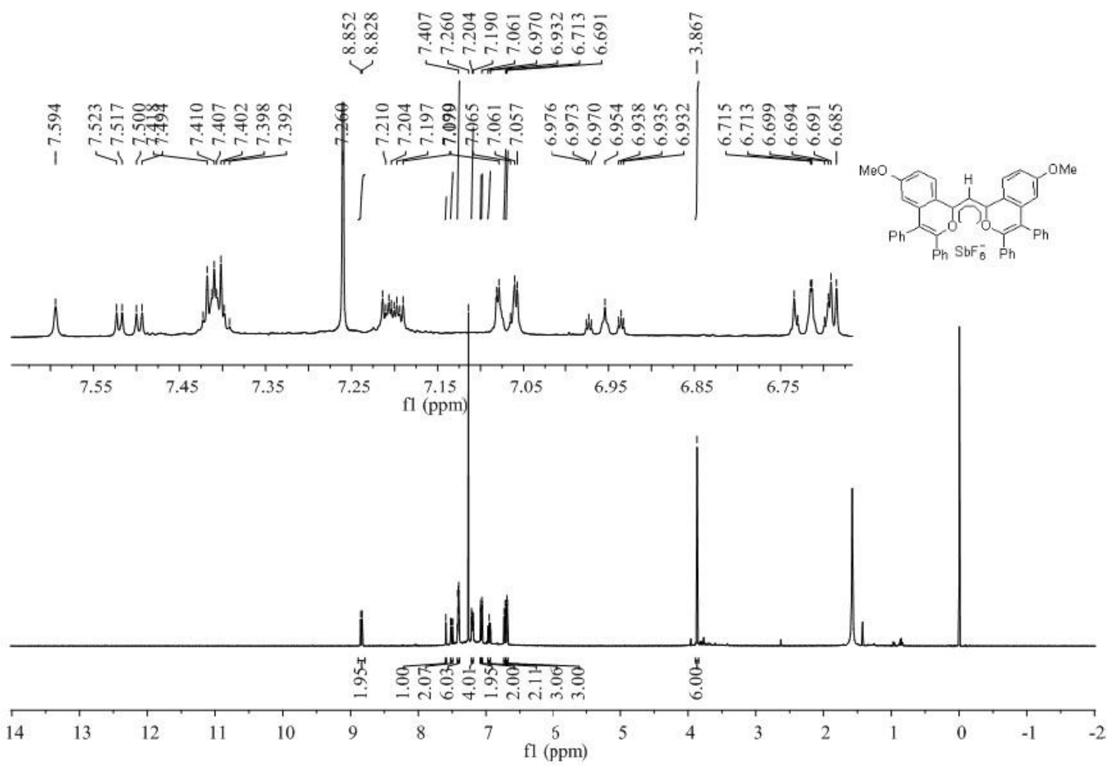

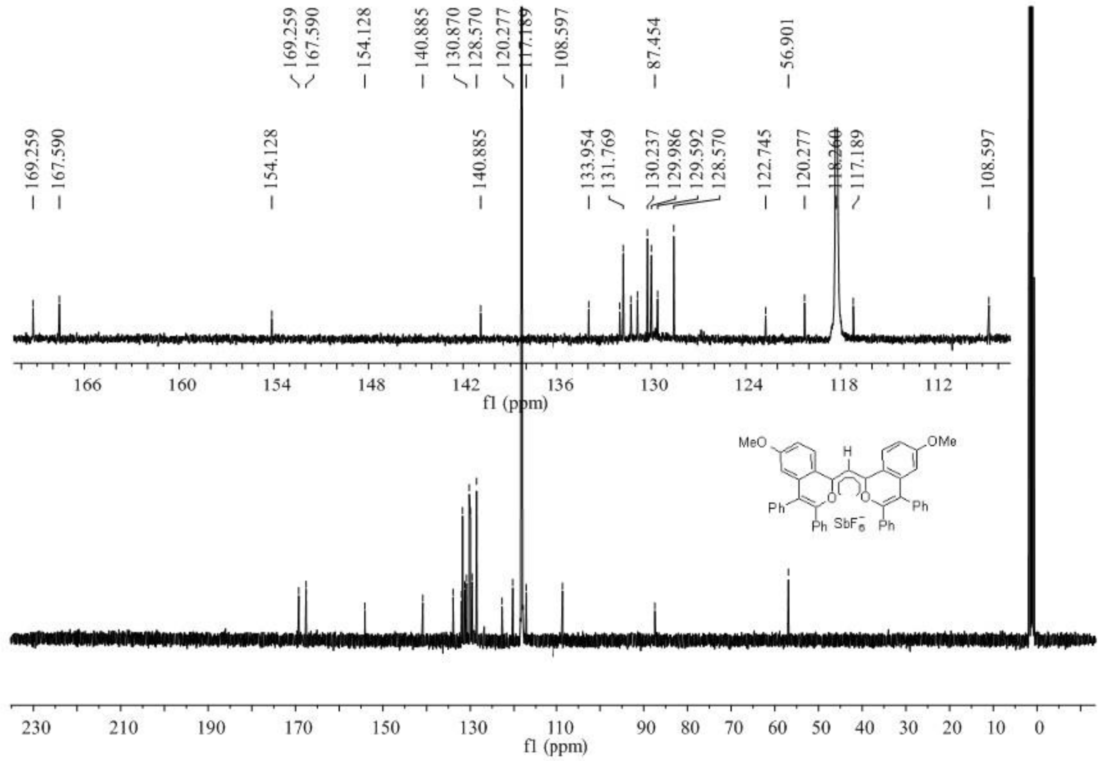

Supplementary Figure 8. ^{1}H NMR and ^{13}C NMR spectra for compound 3aa

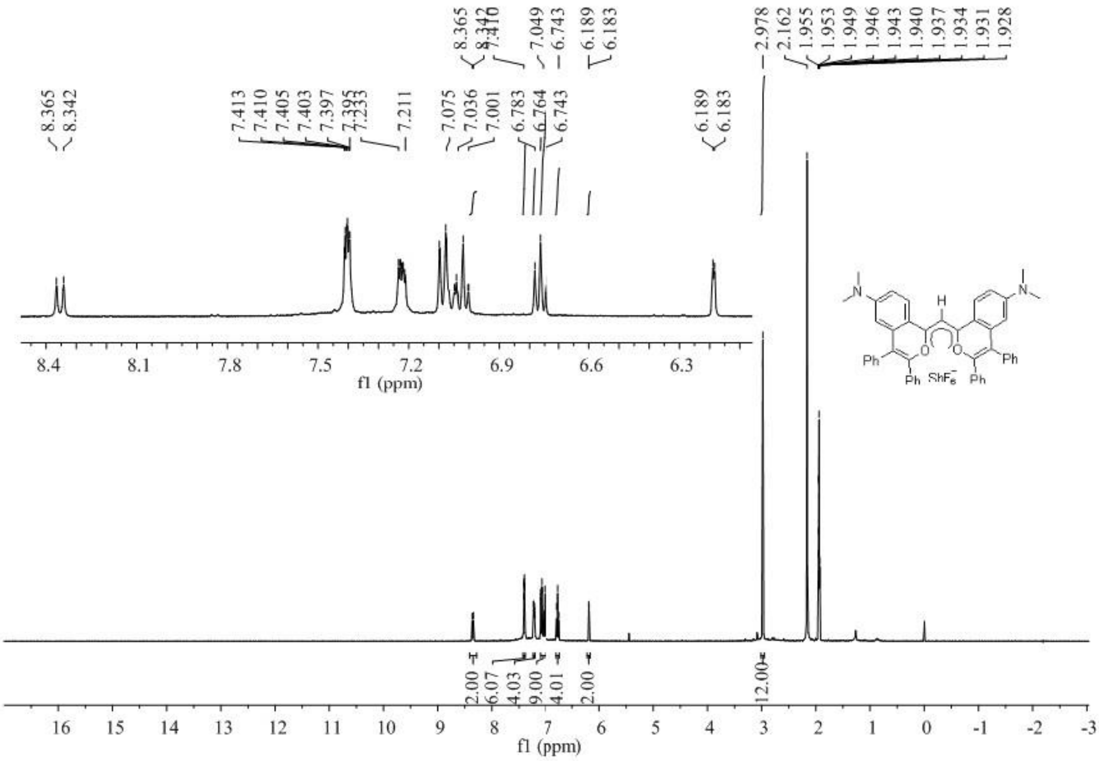

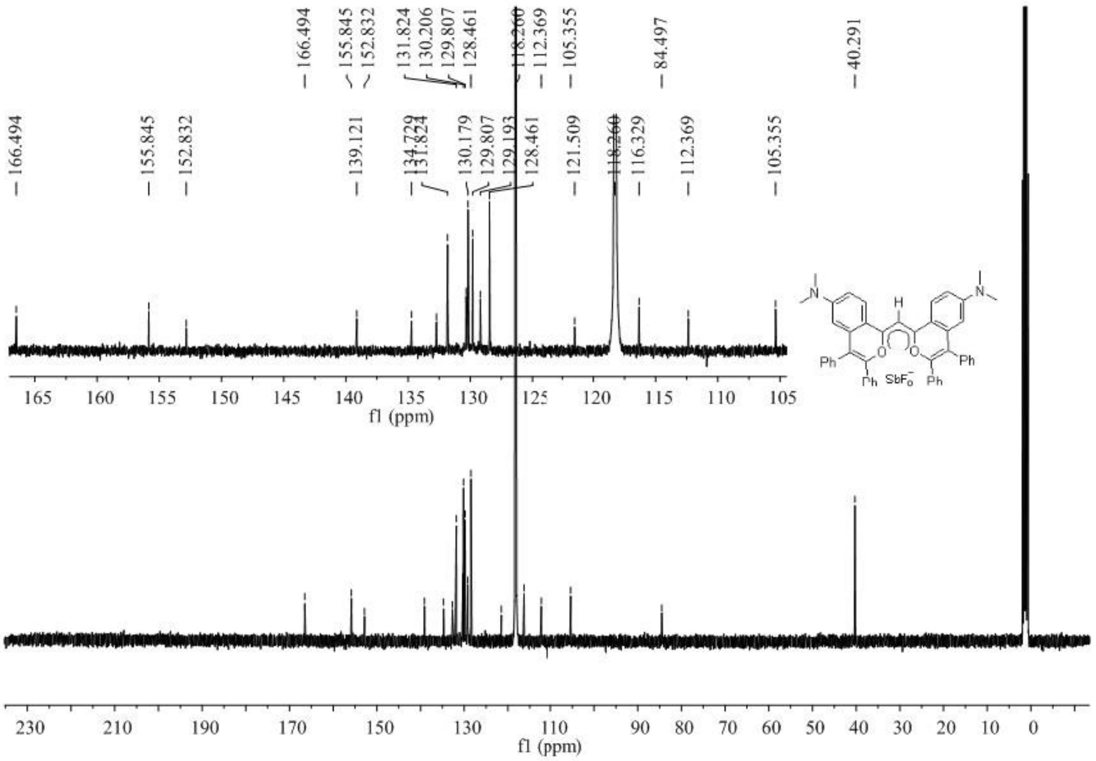

Supplementary Figure 9. ^{1}H NMR and ^{13}C NMR spectra for compound 3ba

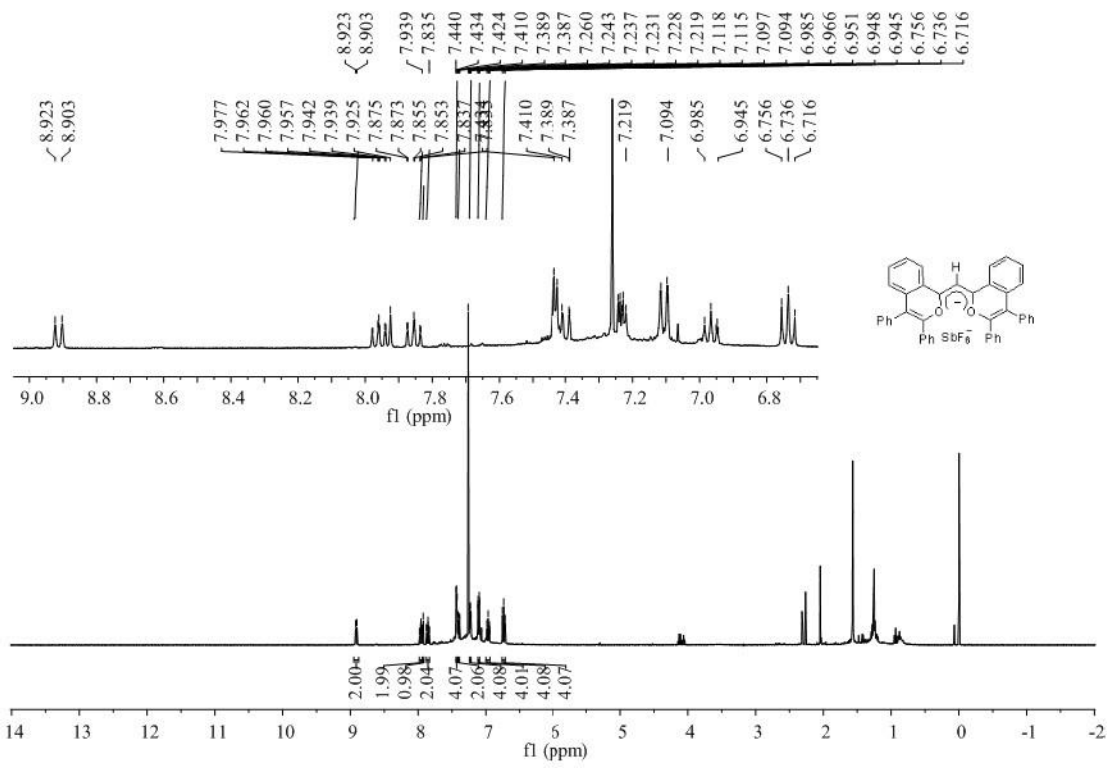

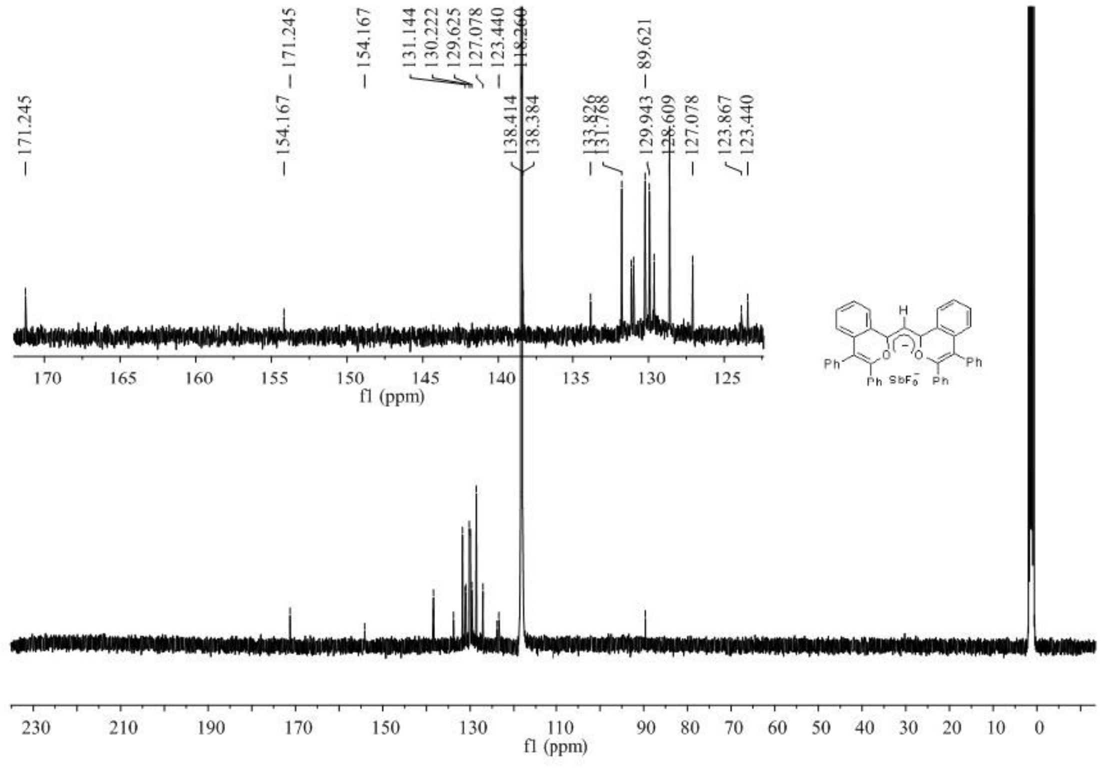

Supplementary Figure 10. ^{1}H NMR and ^{13}C NMR spectra for compound 3ca

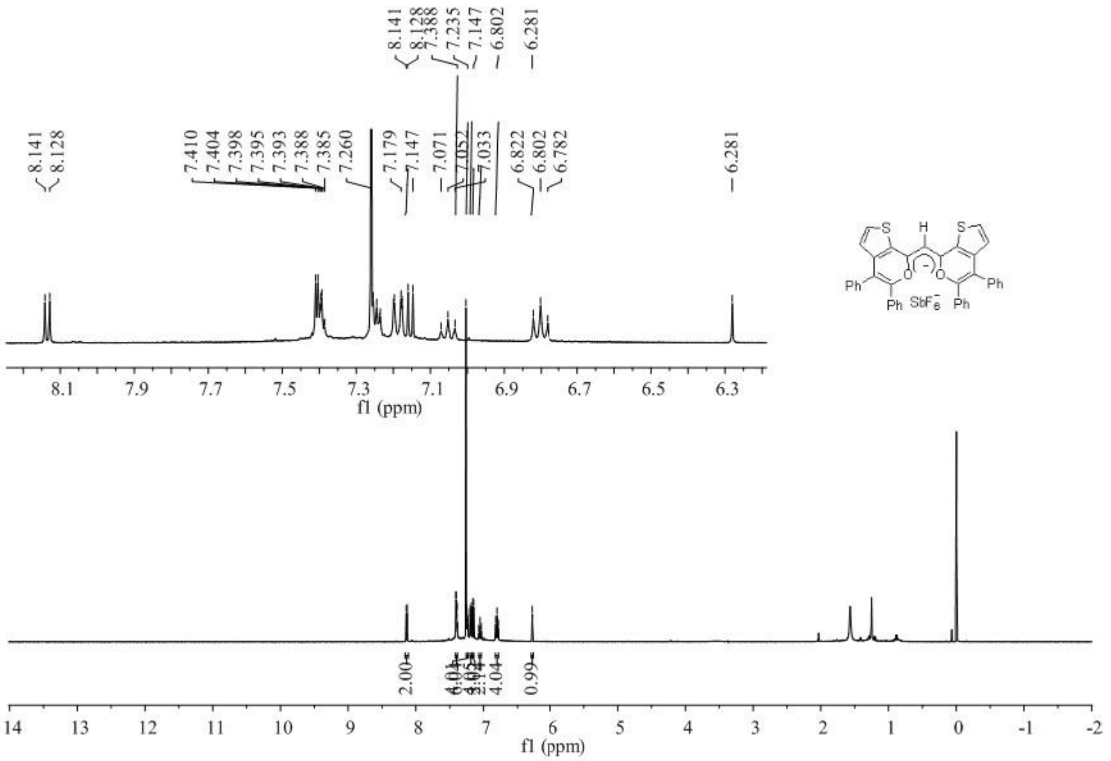

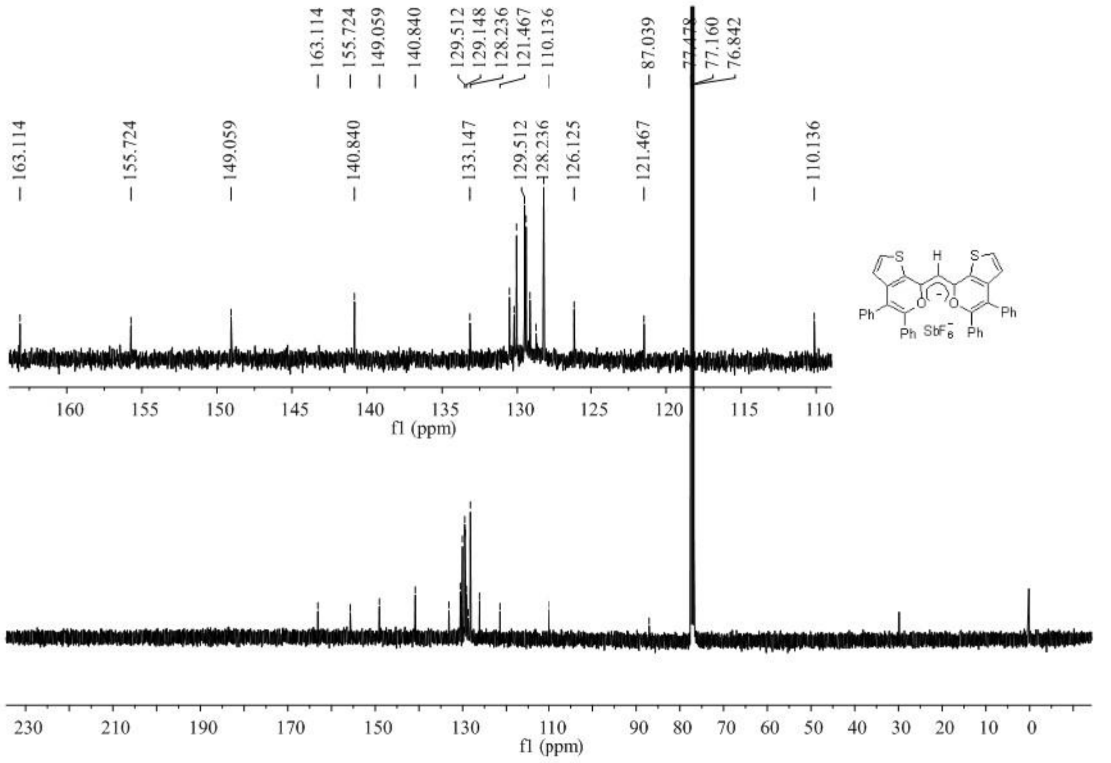

Supplementary Figure 11. ^{1}H NMR and ^{13}C NMR spectra for compound 3da

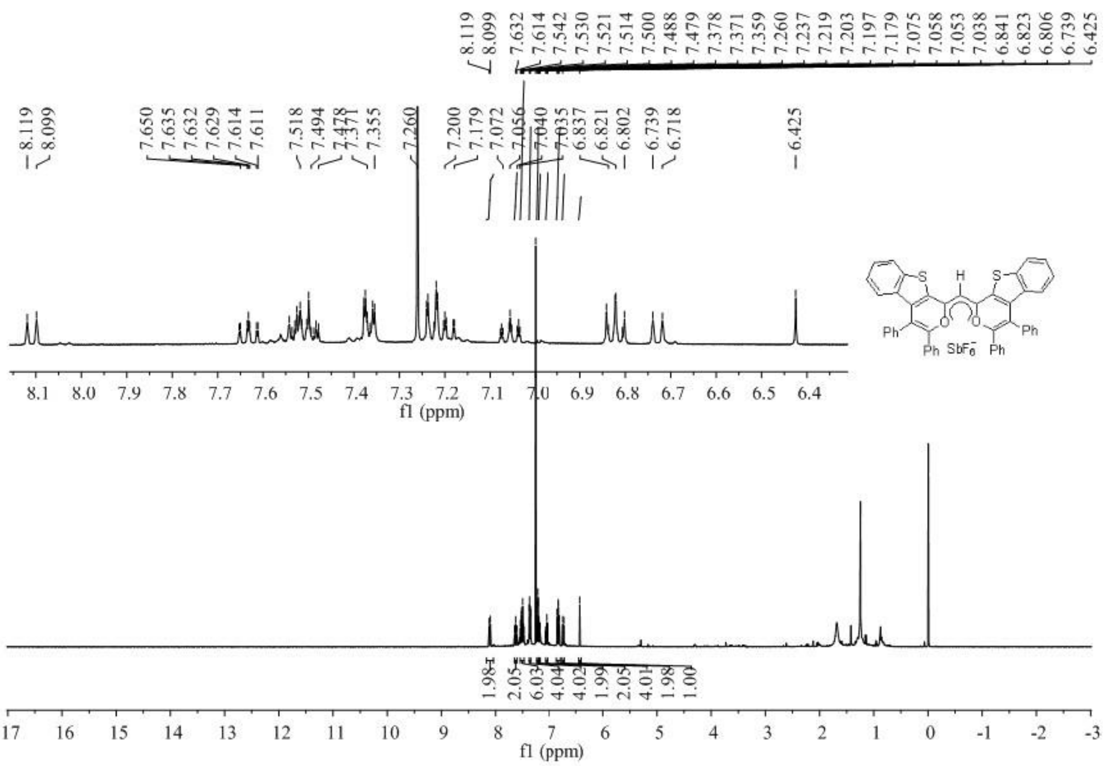

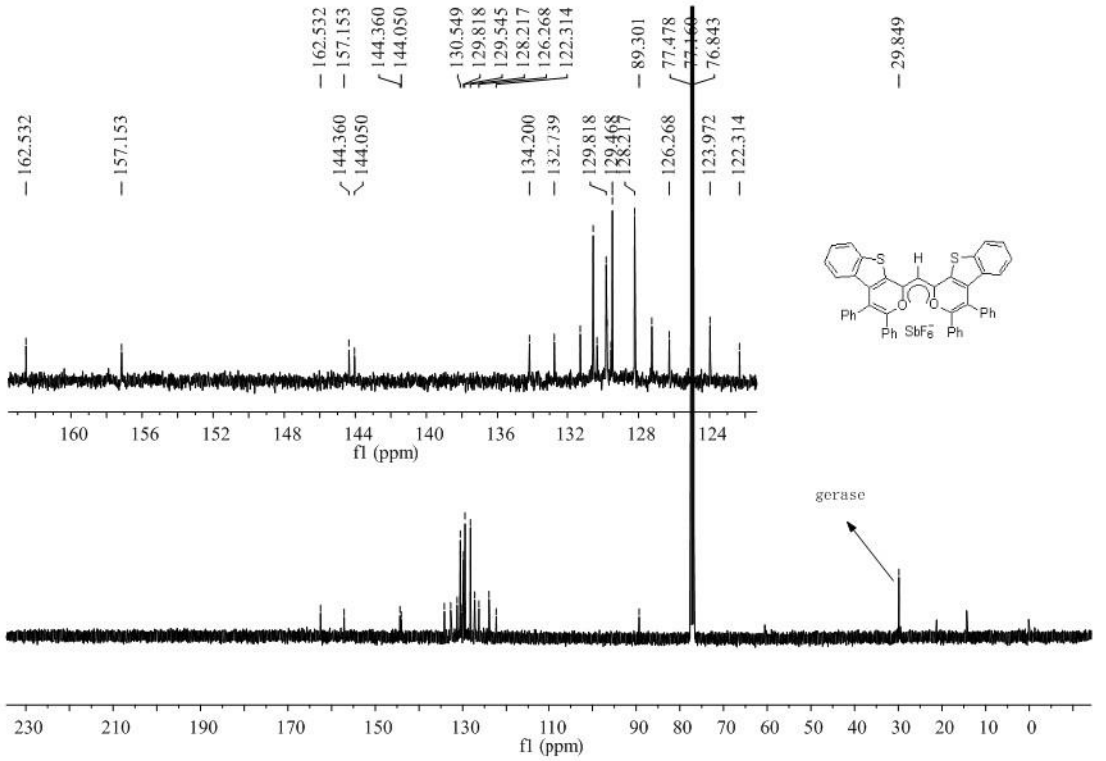

Supplementary Figure 12. ^{1}H NMR and ^{13}C NMR spectra for compound 3ea

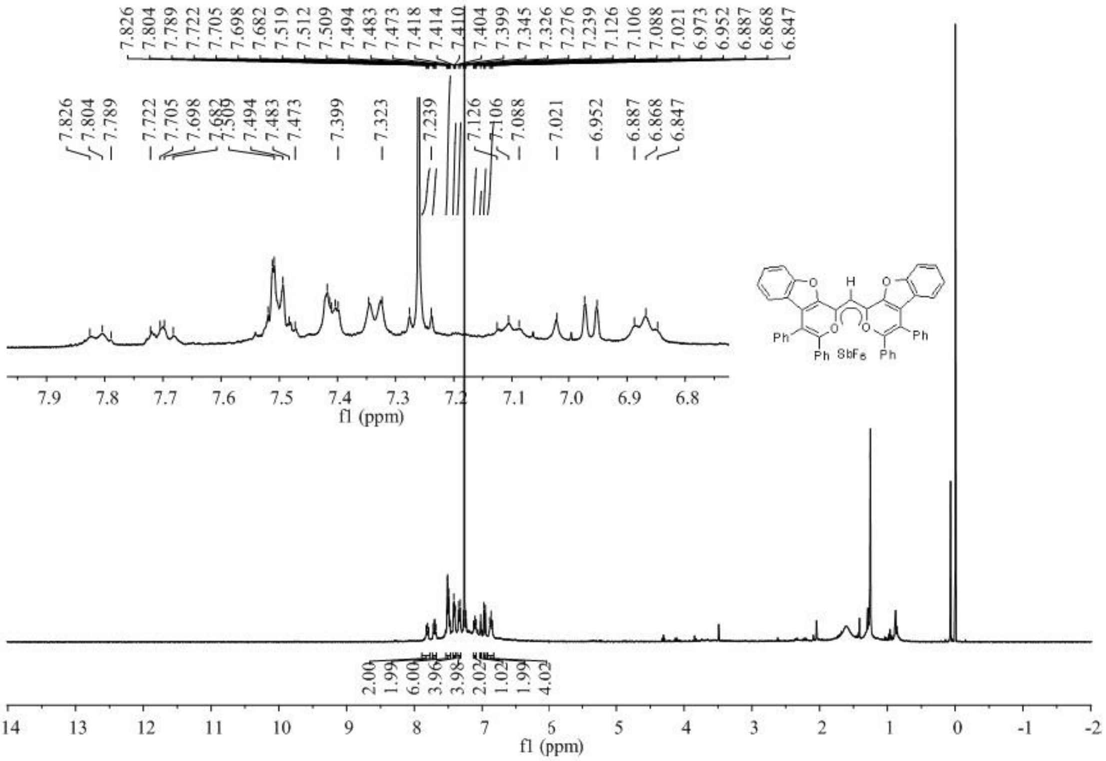

Supplementary Figure 13. ^{1}H NMR spectra for compound 3fa

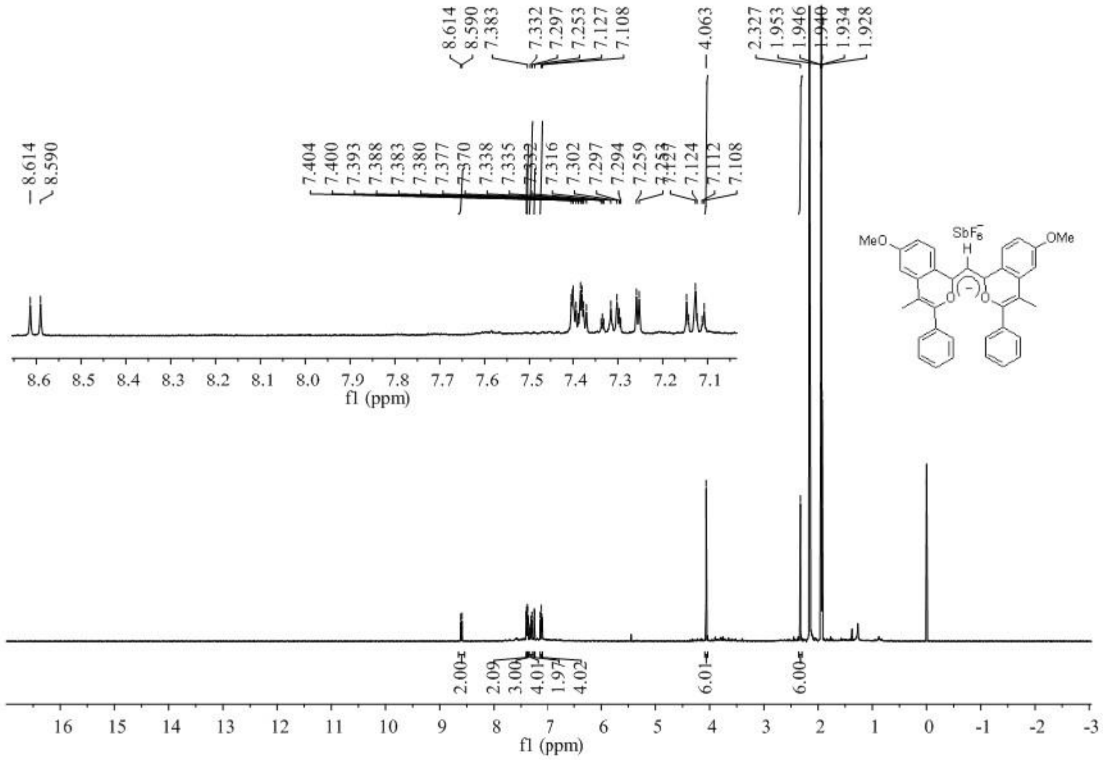

Supplementary Figure 14. ^{1}H NMR spectra for compound 3ad

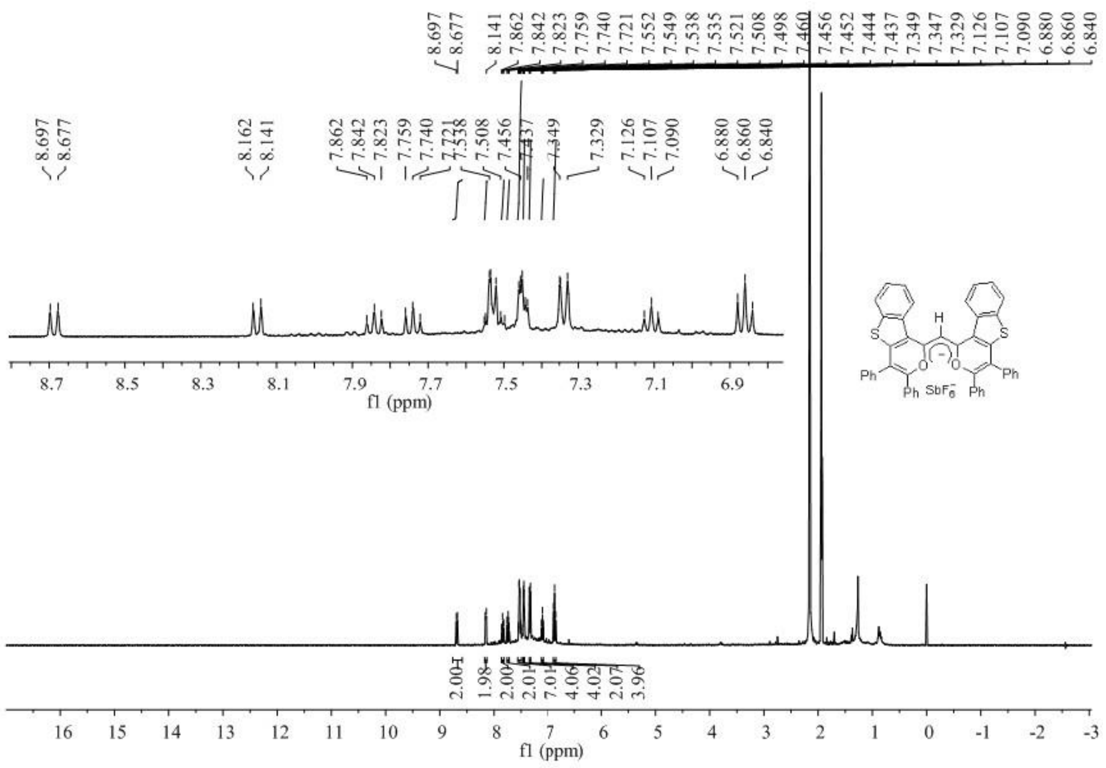

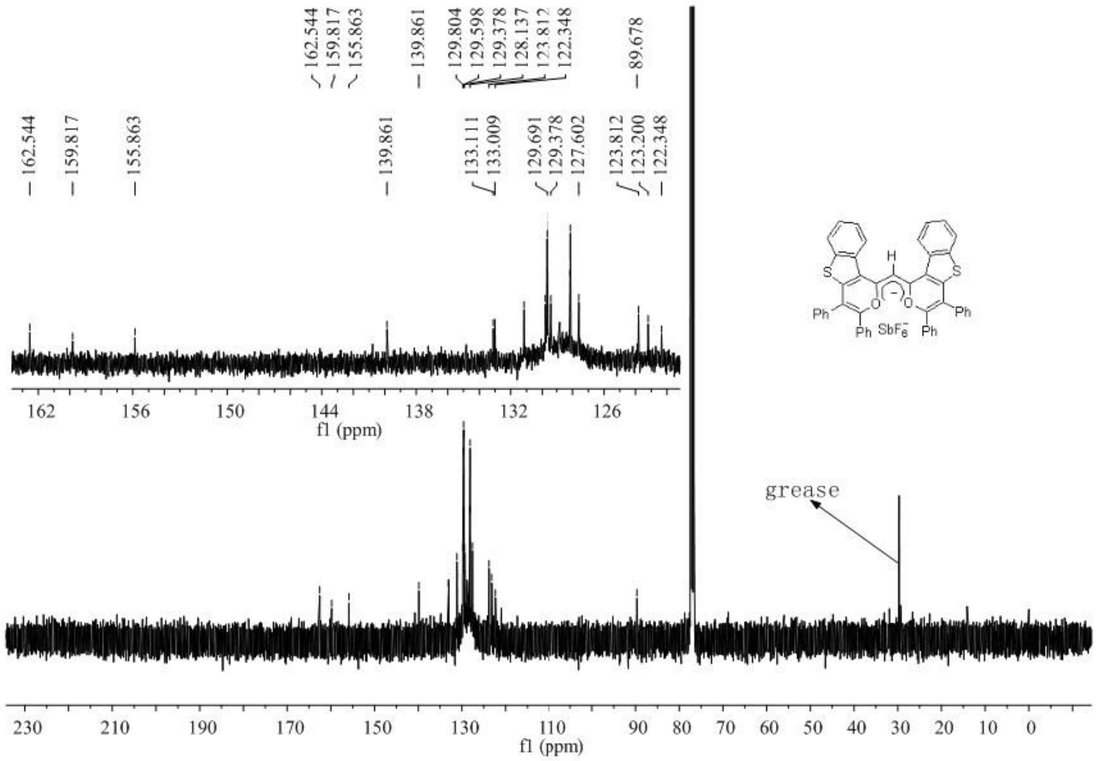

Supplementary Figure 15. ^{1}H NMR and ^{13}C NMR spectra for compound 3ga

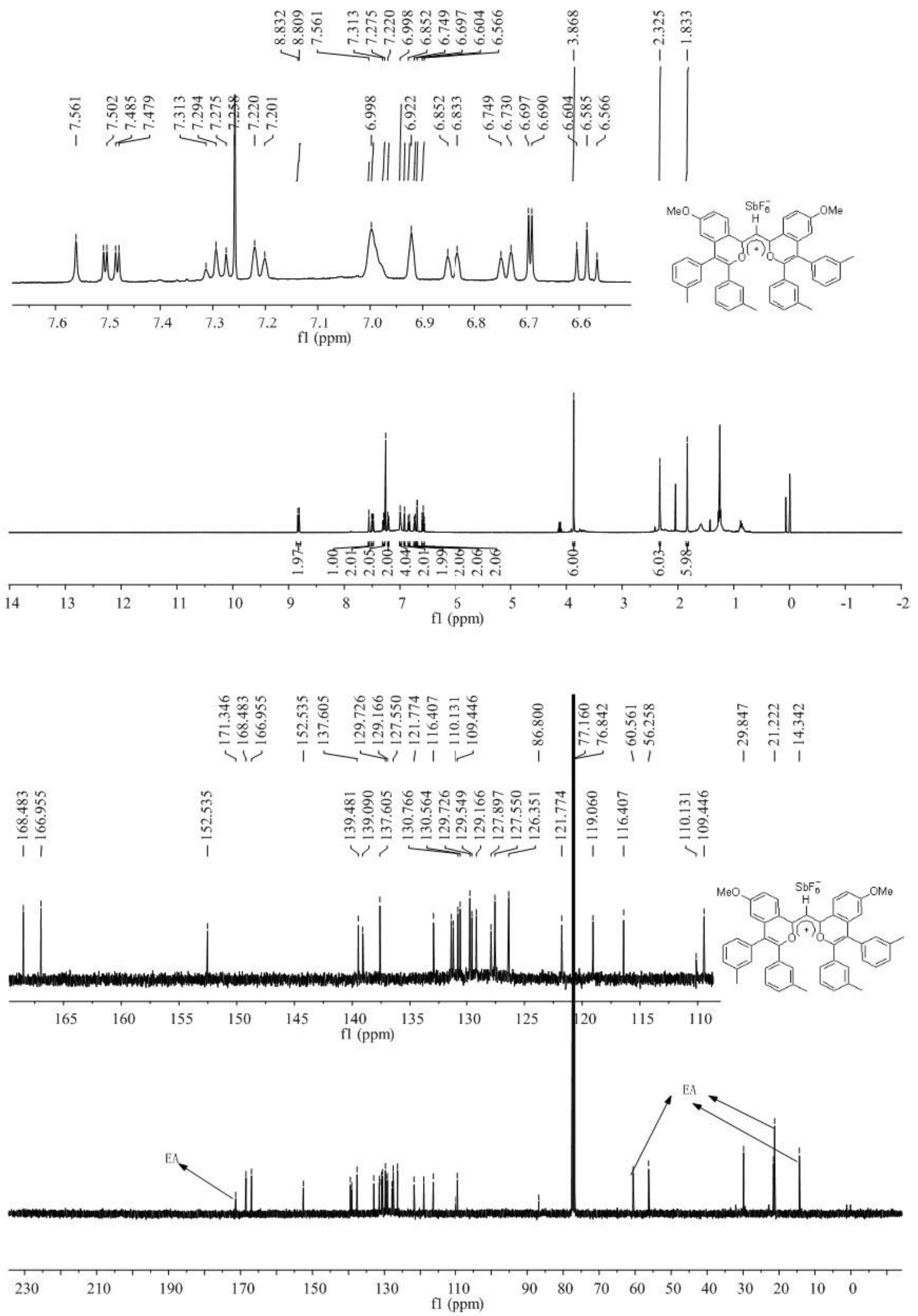

Supplementary Figure 16. ^{1}H NMR and ^{13}C NMR spectra for compound 3ab

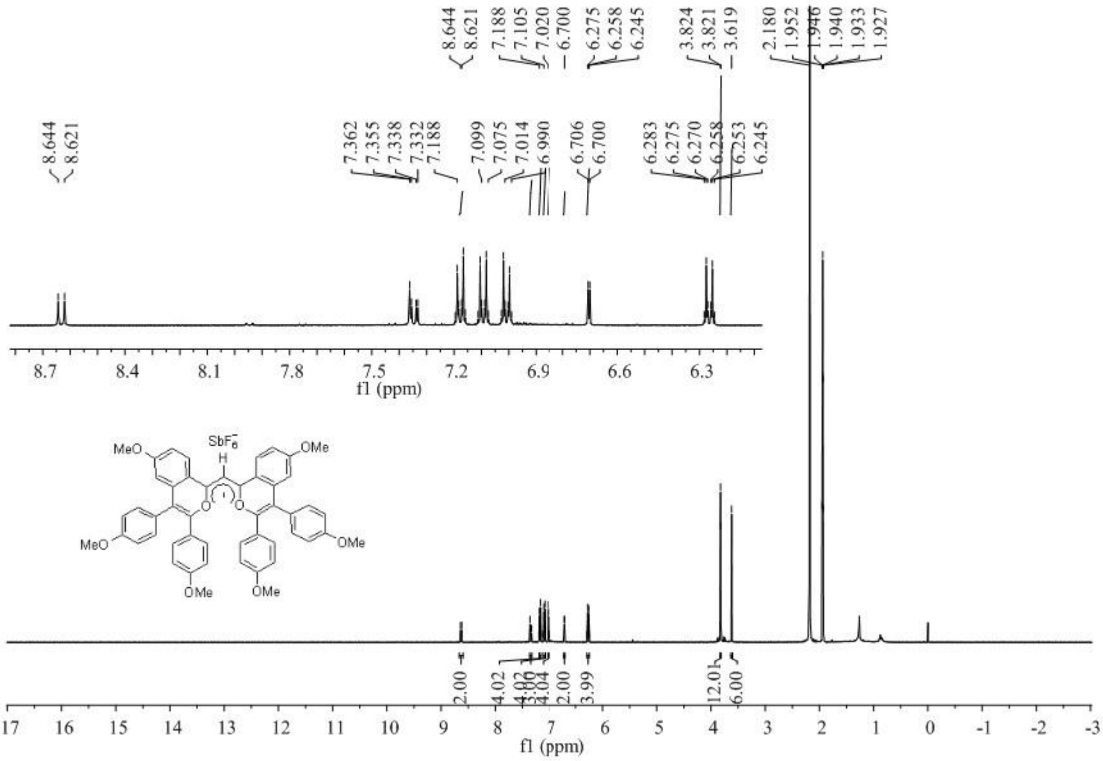

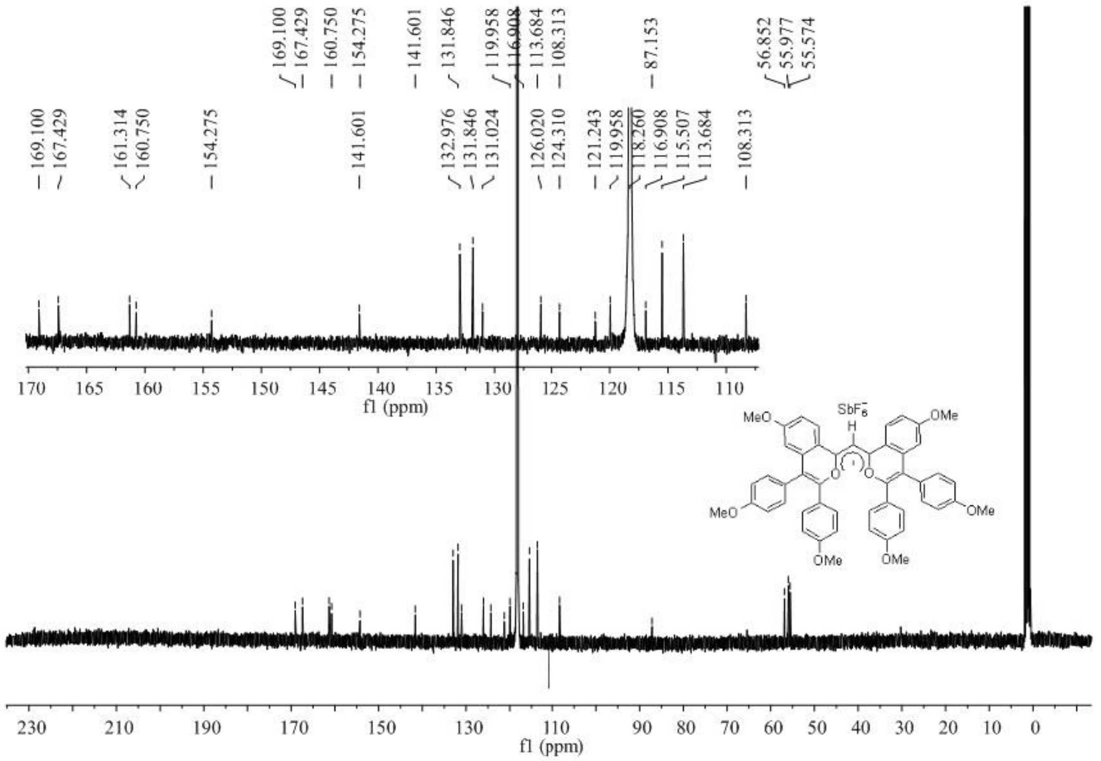

Supplementary Figure 17. ^{1}H NMR and ^{13}C NMR spectra for compound 3ac

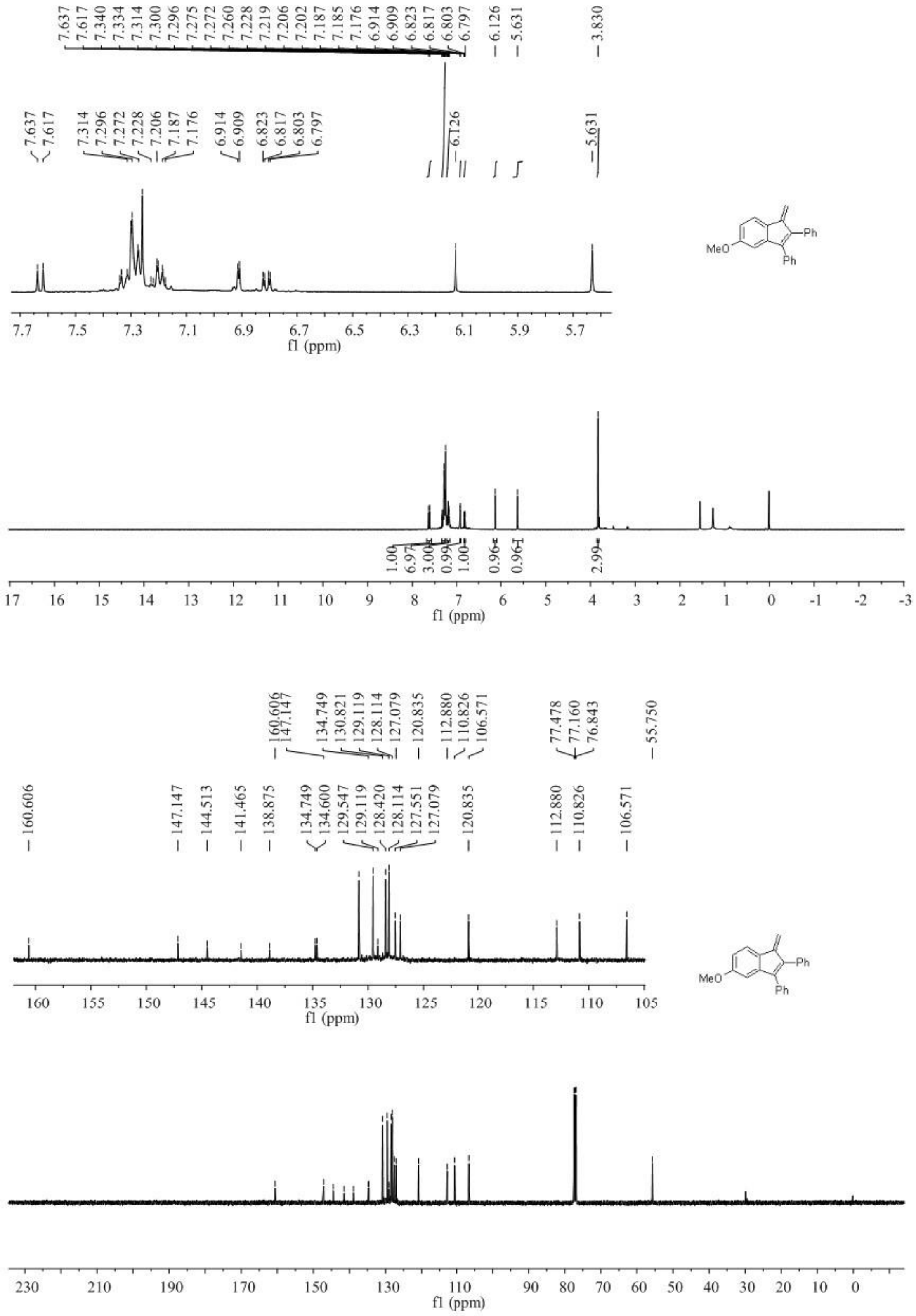

Supplementary Figure 18. ^{1}H NMR and ^{13}C NMR spectra for compound 4aa

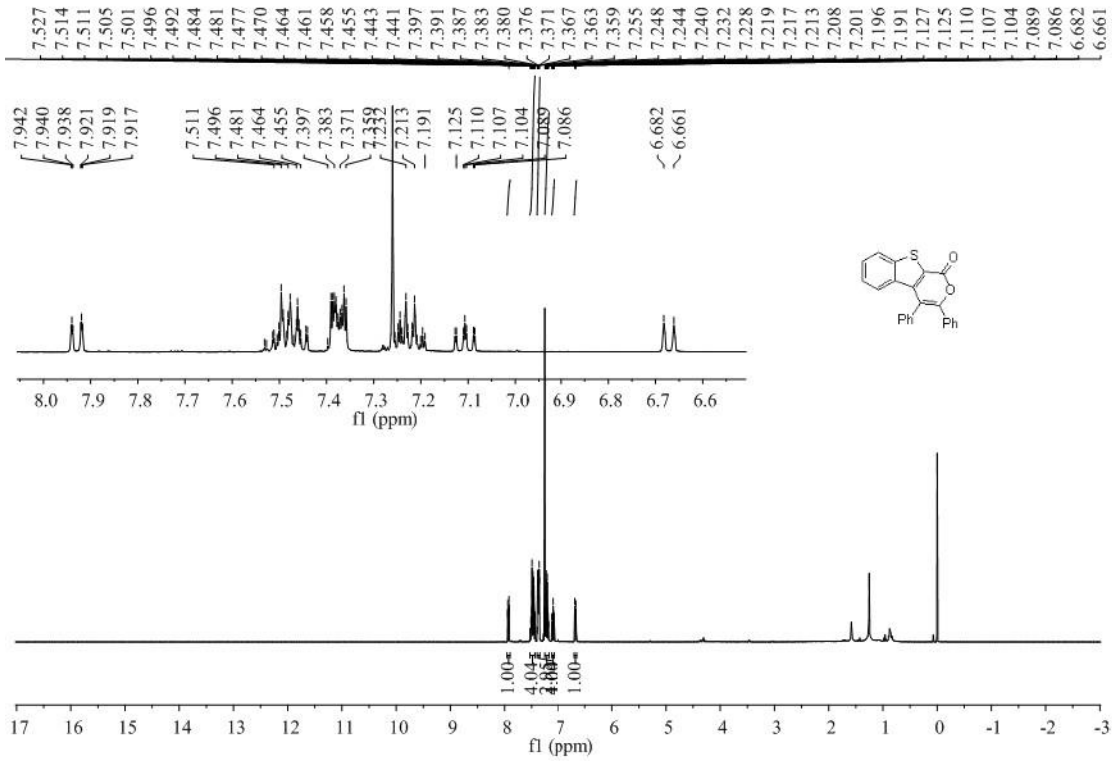

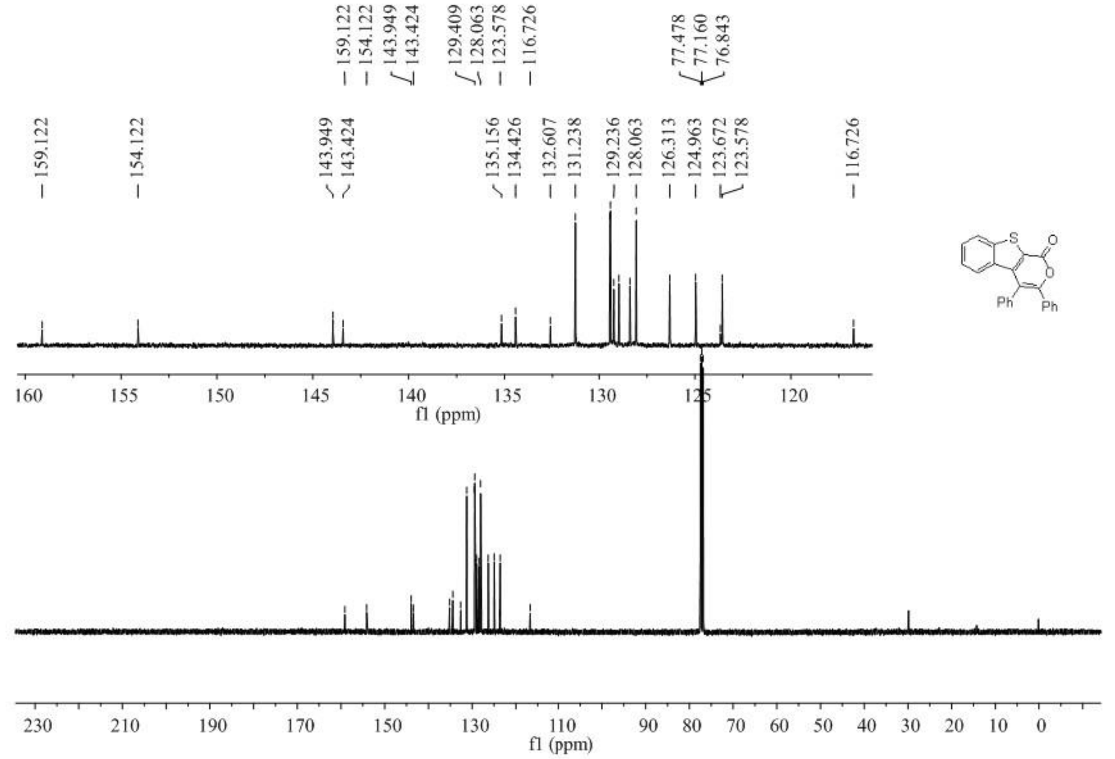

Supplementary Figure 19. ^{1}H NMR and ^{13}C NMR spectra for compound 7ea

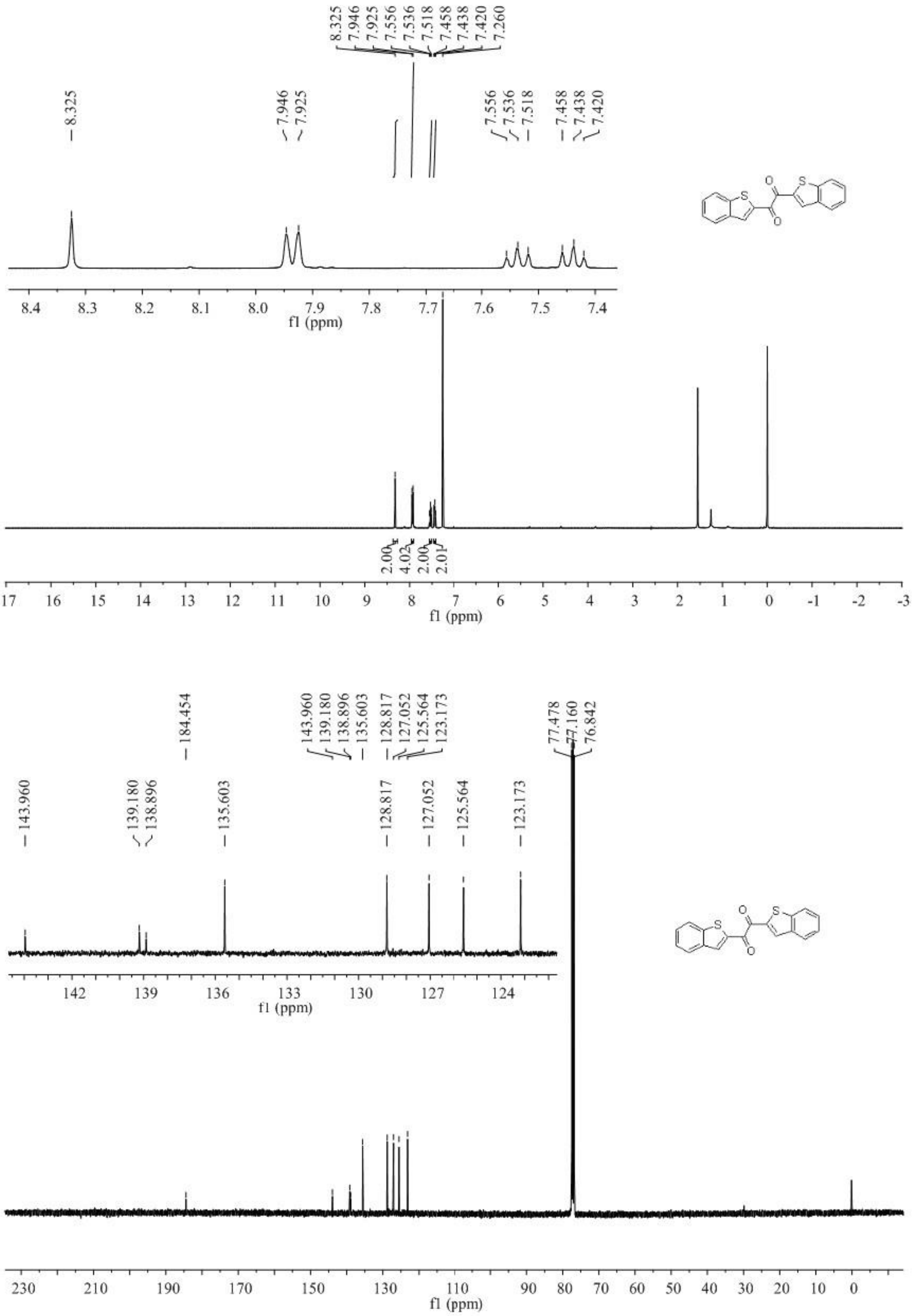

Supplementary Figure 20. ^{1}H NMR and ^{13}C NMR spectra for compound 8ea

Supplementary Tables

Supplementary Table 1 | Optimization for the synthesis of BFFs

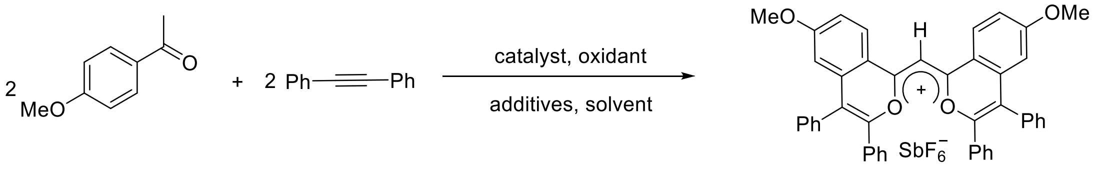

1a **2a** **3aa**

Entry	Metal complex	Oxidant (mmol)	Additive 1(mmol)	Additive 2(mmol)	Solvent (mL)	Yield (%)
1	$[Cp^*RhCl_2]_2/AgSbF_6$	Ag_2O (0.3)	$Cu(OAc)_2 \cdot H_2O$ (0.2)	-	DCE (1.0)	8
2	$[Cp^*RhCl_2]_2$	Ag_2O (0.3)	$Cu(OAc)_2 \cdot H_2O$ (0.2)	-	DCE (1.0)	n.d.
3	$[Cp^*RhCl_2]_2/AgSbF_6$	Ag_2O (0.3)	$Cu(OAc)_2 \cdot H_2O$ (0.2)	$NaSbF_6$ (1.5)	DCE (1.0)	45
4	$[Cp^*RhCl_2]_2/AgSbF_6$	Ag_2O (0.3)	-	$NaSbF_6$ (1.5)	DCE (1.0)	n.d.
5	$[Cp^*RhCl_2]_2/AgSbF_6$	Ag_2O (0.3)	$CuBr_2$ (0.2)	$NaSbF_6$ (1.5)	DCE (1.0)	31
6	$[Cp^*RhCl_2]_2/AgSbF_6$	Ag_2O (0.3)	CuO (0.2)	$NaSbF_6$ (1.5)	DCE (1.0)	n.d.
7	$[Cp^*RhCl_2]_2/AgSbF_6$	Ag_2O (0.3)	CuCl (0.2)	$NaSbF_6$ (1.5)	DCE (1.0)	n.d.
8	$[Cp^*RhCl_2]_2/AgSbF_6$	Ag_2O (0.3)	Cu(OAc) (0.2)	$NaSbF_6$ (1.5)	DCE (1.0)	41
9	$[Cp^*RhCl_2]_2/AgSbF_6$	Ag_2O (0.3)	Cu_2O (0.2)	$NaSbF_6$ (1.5)	DCE (1.0)	n.d.
10	$[Cp^*RhCl_2]_2/AgSbF_6$	Ag_2O (0.3)	$Cu(OAc)_2 \cdot H_2O$ (0.04)	$NaSbF_6$ (1.5)	DCE (1.0)	49
11	$[Cp^*RhCl_2]_2/AgSbF_6$	$Cu(OAc)_2 \cdot H_2O$ (0.3)	$Cu(OAc)_2 \cdot H_2O$ (0.04)	$NaSbF_6$ (1.5)	DCE (1.0)	20
12	$[Cp^*RhCl_2]_2/AgSbF_6$	AgOAc (0.3)	$Cu(OAc)_2 \cdot H_2O$ (0.04)	$NaSbF_6$ (1.5)	DCE (1.0)	45
13	$[Cp^*RhCl_2]_2/AgSbF_6$	Ag_2CO_3 (0.3)	$Cu(OAc)_2 \cdot H_2O$ (0.04)	$NaSbF_6$ (1.5)	DCE (1.0)	55
14	$[Cp^*RhCl_2]_2/AgSbF_6$	O_2	$Cu(OAc)_2 \cdot H_2O$ (0.04)	$NaSbF_6$ (1.5)	DCE (1.0)	n.d.
15	$[Cp^*RhCl_2]_2/AgSbF_6$	Ag_2CO_3 (0.3)	$Cu(OAc)_2 \cdot H_2O$ (0.04)	$NaSbF_6$ (1.5)	DCE (0.5)	63
16	$[Cp^*RhCl_2]_2/AgSbF_6$	Ag_2CO_3 (0.3)	$Cu(OAc)_2 \cdot H_2O$ (0.04)	$NaSbF_6$ (1.5)	DCE (2.0)	49
17	$[Cp^*RhCl_2]_2/AgSbF_6$	Ag_2CO_3 (0.3)	$Cu(OAc)_2 \cdot H_2O$ (0.04)	$NaSbF_6$ (1.5)	toluene (1.0)	n.d.

18	$[Cp^*RhCl_2]_2/AgSbF_6$	Ag_2CO_3 (0.3)	$Cu(OAc)_2 \cdot H_2O$ (0.04)	$NaSbF_6$ (1.5)	THF (1.0)	n.d.
19	$[Cp^*RhCl_2]_2/AgSbF_6$	Ag_2CO_3 (0.3)	$Cu(OAc)_2 \cdot H_2O$ (0.04)	$NaSbF_6$ (1.5)	1,4-dioxane (1.0)	n.d.
20	$[Cp^*RhCl_2]_2/AgSbF_6$	Ag_2CO_3 (0.3)	$Cu(OAc)_2 \cdot H_2O$ (0.04)	$NaSbF_6$ (1.5)	DMF (1.0)	n.d.
21	$[RuCl_2(p\text{-cymene})]_2/AgSbF_6$	Ag_2CO_3 (0.3)	$Cu(OAc)_2 \cdot H_2O$ (0.04)	$NaSbF_6$ (1.5)	DCE (0.5)	n.d.
22	$[Cp^*IrCl_2]_2/AgSbF_6$	Ag_2CO_3 (0.3)	$Cu(OAc)_2 \cdot H_2O$ (0.04)	$NaSbF_6$ (1.5)	DCE (0.5)	21
23*	$[Cp^*RhCl_2]_2/AgSbF_6$	Ag_2CO_3 (0.3)	$Cu(OAc)_2 \cdot H_2O$ (0.04)	$NaSbF_6$ (1.5)	DCE (0.5)	65
24†	$[Cp^*RhCl_2]_2/AgSbF_6$	Ag_2CO_3 (0.3)	$Cu(OAc)_2 \cdot H_2O$ (0.04)	$NaSbF_6$ (1.5)	DCE (0.5)	73
25‡	$[Cp^*RhCl_2]_2/AgSbF_6$	Ag_2CO_3 (0.3)	$Cu(OAc)_2 \cdot H_2O$ (0.04)	$NaSbF_6$ (1.5)	DCE (0.5)	68

Reaction conditions: **1a** (0.2 mmol), **2a** (0.3 mmol), $[Cp^*RhCl_2]_2$ (5 mol %), $AgSbF_6$ (20 mol %), oxidative, additives and solvent at 150 ℃ under N_2 for 12 h. Isolated yield. *The reaction was carried out at 120 ℃. †The reaction was carried out at 100 ℃. ‡The reaction was carried out at 80 ℃. n.d. = not detected. DCE = 1,2-dichloroethane. THF = tetrahydrofuran. DMF = *N*,*N*-dimethylformamide.

Supplementary Table 2 | The synthesis of 3aa in the presence of TEMPO

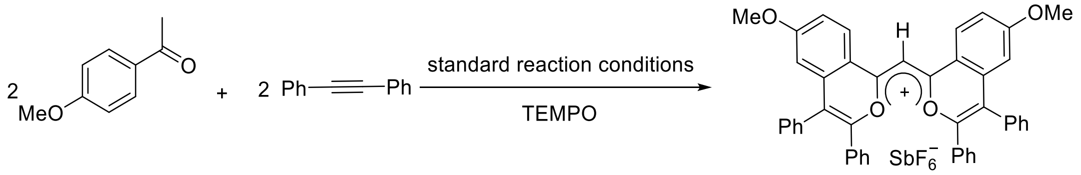

1a **2a** **3aa**

Entry	Radical Scavenger	Equivalent	Yield (%)
1	TEMPO	0.2	52
2	TEMPO	0.5	33
3	TEMPO	1.0	<5

Reaction conditions: **1a** (0.2 mmol), **2a** (0.3 mmol), $[Cp^*RhCl_2]_2$ (5 mol %), $AgSbF_6$ (0.02 mmol), Ag_2CO_3 (0.3 mmol), $Cu(OAc)_2 \cdot H_2O$ (0.04 mmol), $NaSbF_6$ (0.15 mmol), TEMPO and DCE (0.5 mL) at 100 ℃ under N_2 for 12 h. Isolated yield. TEMPO = (2,2,6,6-tetramethylpiperidin-1-yl)oxyl.

Supplementary Table 3 | Photophysical data of the products

Compound	λ_{abs}/nm	λ_{ex}/nm	λ_{em}/nm	Stokes shift (cm^{-1})	$\varepsilon/M^{-1}cm^{-1}$	Φ_F

**3aa**	563	565	591	842	21775	0.60
**3ba**	582	582	597	432	15700	0.50
**3ca**	590	593	626	975	6525	0.46
**3da**	574	574	608	975	3800	0.64
**3ea**	617	617	656	964	4650	0.59
**3fa**	625	625	660	823	7675	0.63
**3ga**	586	587	618	884	3850	0.51
**3ab**	567	569	596	858	23400	0.60
**3ac**	585	585	631	1246	17825	0.49
**3ad**	553	553	577	752	16275	0.44

Absorption and emission maximum in CH_2Cl_2 at 40.0 μM.

Supplementary Table 4 | Crystal data and structure refinement for 3aa

Identification code	**3aa**
Empirical formula	$C_{45}H_{33}F_6O_4Sb$
Formula weight	873.46
Temperature/K	293.15
Crystal system	monoclinic
Space group	I2/a
a/Å	21.5229(9)
b/Å	17.5061(6)
c/Å	24.1902(8)
α/°	90
β/°	92.394(4)
γ/°	90
Volume/Å^3	9106.5(6)
Z	8
ρ_{calc}g/cm^3	1.274
μ/mm^{-1}	0.667
F(000)	3520.0
Crystal size/mm^3	0.25 × 0.25 × 0.2
Radiation	MoKα (λ = 0.71073)
2Θ range for data collection/°	6.002 to 52.746
Index ranges	$-26 \leq h \leq 18$, $-21 \leq k \leq 19$, $-30 \leq l \leq 28$
Reflections collected	19030
Independent reflections	9302 [R_{int} = 0.0259, R_{sigma} = 0.0617]
Data/restraints/parameters	9302/0/507
Goodness-of-fit on F^2	0.763
Final R indexes [I>=2σ (I)]	R_1 = 0.0480, wR_2 = 0.1264
Final R indexes [all data]	R_1 = 0.0970, wR_2 = 0.1496
Largest diff. peak/hole / e Å^{-3}	0.53/-0.46

Supplementary Table 5 | Crystal data and structure refinement for 3ad

Identification code	**3ad**
Empirical formula	$C_{35}H_{29}F_6O_4Sb$
Formula weight	749.33
Temperature/K	293.15
Crystal system	monoclinic
Space group	I2/a
a/Å	14.9507(13)
b/Å	24.1146(18)
c/Å	17.9754(14)
α/°	90
β/°	103.720(8)
γ/°	90
Volume/Å^3	6295.8(9)
Z	8
ρ_{calc}g/cm^3	1.581
μ/mm^{-1}	0.950
F(000)	3008.0
Crystal size/mm^3	0.35 × 0.1 × 0.05
Radiation	MoKα (λ = 0.71073)
2Θ range for data collection/°	5.792 to 52.742
Index ranges	$-18 \le h \le 18$, $-26 \le k \le 30$, $-15 \le l \le 22$
Reflections collected	15371
Independent reflections	6423 [R_{int} = 0.1096, R_{sigma} = 0.1377]
Data/restraints/parameters	6423/0/426
Goodness-of-fit on F^2	1.141
Final R indexes [I>=2σ (I)]	R_1 = 0.1378, wR_2 = 0.3802
Final R indexes [all data]	R_1 = 0.2212, wR_2 = 0.4322
Largest diff. peak/hole / e Å^{-3}	1.05/-1.63

Supplementary Methods

I. General remarks

NMR spectra were recorded on a Varian Inova 400 spectrometer. The ^{1}H NMR (400 MHz) chemical shifts were recorded relative to $CDCl_3$ or CD_3CN as the internal reference ($CDCl_3$: δ_H = 7.26 ppm; CD_3CN: δ_H = 1.94 ppm). The ^{13}C NMR (100 MHz) chemical shifts were given using $CDCl_3$ or CD_3CN as the internal standard ($CDCl_3$:

δ_C = 77.16 ppm; CD_3CN: δ_C = 118.26 ppm). High-resolution mass spectra (HRMS) were obtained with a Shimadzu LCMS-IT-TOF (ESI) or a Waters-Q-TOF-Premier (ESI). X-Ray single-crystal diffraction data were collected on an Oxford Xcalibur E single crystal diffraction. UV/Vis spectra experiments were conducted on a HITACHI U-2910. Fluorescence spectra were measured on a Horiba Jobin Yvon-Edison Fluoromax-4 fluorescence spectrometer with a calibrated integrating sphere system.

Unless otherwise noted, all reagents were obtained from commercial suppliers and used without further purification. $[Cp^*RhCl_2]_2$ were prepared according to the literature procedures.[1] The solvents were purified and dried using an Innovative Technology PS-MD-5 Solvent Purification System. $RhCl_3 \cdot xH_2O$ were purchased from Shanxi Kaida Chemical Engineering (China) CO., Ltd. Unless otherwise noted, all reactions were performed with dry solvents under an atmosphere of nitrogen in glassware with standard vacuum-line techniques.

II. Optimization of the reaction conditions for the synthesis of BFFs

A Schlenk tube with a magnetic stir bar was charged with metal complex (5.0 µmol, 5.0 mol%), $AgSbF_6$ (20 µmol, 20 mol%), oxidative, additives, 4-methoxyacetophenone (30.0 mg, 0.2 mmol), diphenylacetylene (53.4 mg, 0.3 mmol), and solvent under an N_2 atmosphere. The resulting solution was stirred at room temperature for 10 min and then at the indicated temperature for 12 h. Subsequently, it was diluted with 5 mL of dichloromethane. The mixture was evaporated under reduced pressure and the residue was absorbed to small amounts of silica gel. The purification was performed by column chromatography on silica gel (petroleum ether /ethyl acetate = 1:1, v/v, then petroleum ether /dichloromethane /ethyl acetate = 1:1:4, v/v/v, then dichloromethane/methanol = 20:1, v/v) to provide **3aa** (Supplementary Table 1)**.**

III. General procedure for the synthesis of BFFs

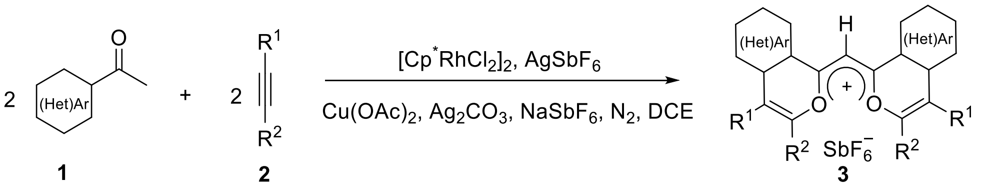

A Schlenk tube with a magnetic stir bar was charged with $[Cp^{*}RhCl_2]_2$ (0.005 mmol), $AgSbF_6$ (0.02 mmol), Ag_2CO_3 (0.3 mmol), $Cu(OAc)_2$ H_2O (0.04 mmol), $NaSbF_6$ (0.15 mmol), arylketone (0.2 mmol), alkyne (0.3 mmol), and DCE (0.5 mL) under an N_2 atmosphere. The resulting solution was stirred at room temperature for 10 min and then at the indicated temperature for the indicated time. The resulting solution was cooled to ambient temperature, diluted with 10 mL of dichloromethane. The obtained organic extracts was evaporated under reduced pressure and the residue was absorbed into small amounts of silica gel. Purification was performed by column chromatography on silica gel to provide product **3**.

IV. General procedure for the preparation of arylketones

Arylketones **1a**, **1b**, **1c**, **1d**, **1e** and **1f** were purchased from commercial suppliers and used without further purification. For the preparation of **1g**, the following procedure was used (Supplementary Figure 1).[2]

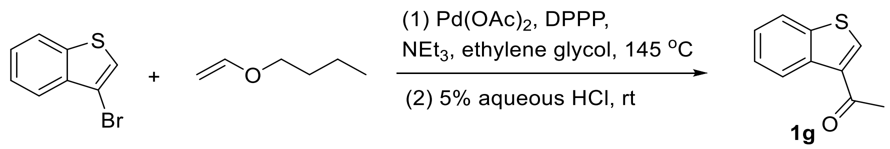

A Schlenk tube with a magnetic stir bar was charged with 3-bromobenzo[*b*]thiophene (1.0 mmol), arylboronic acid (1.4 mmol), $Pd(OAc)_2$ (11.0 mg, 0.05 mmol), DPPP (41.0 mg, 0.1 mmol), and ethylene glycol (2.0 mL) under an N_2 atmosphere at room temperature. Following degassing three times, *n*-Butyl vinyl ether (3.0 mmol) and NEt_3 (2.5 mmol) were sequentially added into the reaction mixture. The obtained mixture was stirred at 145 ℃ for 2 h. Then the tube was cooled to room temperature and aqueous HCl (5%, 5 mL) was added into the mixture. The mixture was stirred at room temperature for 0.5 h. Purification was conducted by column chromatography on silica gel to provide the desired product **1g**.

V. General procedure for the preparation of alkynes

Alkynes **2a** and **2d** were purchased from the commercial suppliers and were used without further purification. Alkynes **2b** and **2c** were synthesized according to the reported literature procedures (Supplementary Figure 2).[3]

VI. Preparation and characterization of the described substances

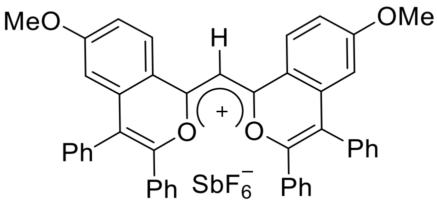

(*Z*)-6-Methoxy-1-((6-methoxy-3,4-diphenyl-1*H*-isochromen-1-ylidene)methyl)-3,4-diphenylisochromenylium hexafluoroantimonate (3aa)

Following the general procedure. 4-Methoxyacetophenone (30.0 mg, 0.2 mmol), and diphenylacetylene (53.4 mg, 0.3 mmol) were used. The reaction was carried at 100 ℃ for 12 h. Purification via column chromatography on silica gel (petroleum ether /ethyl acetate = 1:1, v/v, then petroleum ether /dichloromethane /ethyl acetate = 1:1:4, v/v/v, then dichloromethane/methanol = 20:1, v/v) afforded **3aa** as a dark purple solid (63.7 mg, 73% yield). ^{1}H NMR ($CDCl_3$, 400 MHz): δ (ppm) 8.84 (d, J = 9.6 Hz, 2H), 7.59 (s, 1H), 7.52-7.49 (m, 2H), 7.42-7.39 (m, 6H), 7.21-7.19 (m, 4H), 7.081-7.079 (m, 2H), 7.07-7.06 (m, 2H), 6.98-6.93 (m, 2H), 6.73-6.71 (m, 3H), 6.70-6.69 (m, 3H), 3.87 (s, 6H). ^{13}C NMR (CD_3CN, 100 MHz): δ (ppm) 169.26, 167.59, 154.13, 140.89, 133.95, 131.99, 131.77, 131.29, 130.87, 130.24, 129.99, 129.59, 128.57, 122.75, 120.28, 117.19, 108.60, 87.45, 56.90. HRMS (ESI$^+$): calcd for $C_{45}H_{33}O_4^+$, 637.2373; found 637.2370.

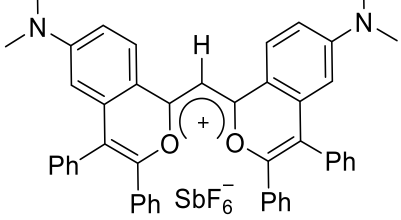

(*Z*)-6-(Dimethylamino)-1-((6-(dimethylamino)-3,4-diphenyl-1*H*-isochromen-1-yli

dene)methyl)-3,4-diphenylisochromenylium hexafluoroantimonate (3ba)

Following the general procedure. 1-(4-(Dimethylamino)phenyl)ethan-1-one (32.7 mg, 0.2 mmol), and diphenylacetylene (53.4 mg, 0.3 mmol) were used. The reaction was carried at 75 ℃ for 4 h. Purification via column chromatography on silica gel (petroleum ether /ethyl acetate = 1:1, v/v, then petroleum ether /dichloromethane /ethyl acetate = 1:1:4, v/v/v, then dichloromethane/methanol = 20:1, v/v) afforded **3ba** as a dark purple solid (34.9 mg, 39% yield). ^{1}H NMR (CD_3CN, 400 MHz): δ (ppm) 8.35 (d, J = 9.2 Hz, 2H), 7.41-7.40 (m, 6H), 7.23-7.21 (m, 4H), 7.10-7.00 (m, 9H), 6.76 (t, J = 8.0 Hz, 4H), 6.19 (d, J = 2.4 Hz, 2H), 2.98 (s, 12H). ^{13}C NMR (CD_3CN, 100 MHz): δ (ppm) 166.49, 155.84, 152.83, 139.12, 134.73, 132.72, 131.82, 130.34, 130.20, 130.18, 129.81, 129.19, 128.46, 121.51, 116.33, 112.36, 105.35, 84.50, 40.29. HRMS (ESI$^+$): calcd for $C_{47}H_{39}N_2O_2^+$, 663.3006; found 663.3004.

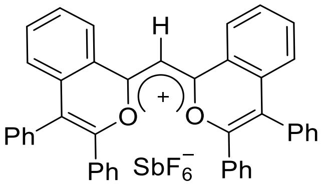

(*Z*)-1-((3,4-Diphenyl-1*H*-isochromen-1-ylidene)methyl)-3,4-diphenylisochromenylium hexafluoroantimonate (3ca)

Following the general procedure. Acetophenone (24.0 mg, 0.2 mmol), and diphenylacetylene (53.4 mg, 0.3 mmol) were used. The reaction was carried at 150 ℃ for 12 h. Purification via column chromatography on silica gel (petroleum ether /ethyl acetate = 1:1, v/v, then petroleum ether /dichloromethane /ethyl acetate = 1:1:4, v/v/v, then dichloromethane/methanol = 20:1, v/v) afforded **3ca** as a dark purple solid (32.5 mg, 40% yield). ^{1}H NMR ($CDCl_3$, 400 MHz): δ (ppm) 8.91 (d, J = 8.0 Hz, 2H), 7.98-7.94 (m, 2H), 7.93 (s, 1H), 7.88-7.84 (m, 2H), 7.44-7.42 (m, 4H), 7.41-7.39 (m, 2H), 7.24-7.22 (m, 4H), 7.12-7.09 (m, 4H), 6.99-6.95 (m, 4H), 6.76-6.72 (m, 4H). ^{13}C NMR ($CDCl_3$, 100 MHz): δ (ppm) 171.25, 154.17, 138.41, 138.38, 133.83, 131.77, 131.14, 130.98, 130.22, 129.94, 129.63, 128.61, 127.08, 123.87, 123.44, 89.62. HRMS (ESI$^+$): calcd for $C_{43}H_{29}O_2^+$, 577.2162; found 577.2163.

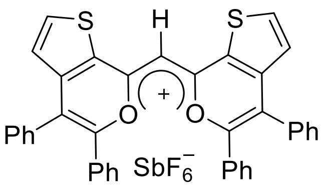

(*Z*)-7-((4,5-Diphenyl-7*H*-thieno[2,3-c]pyran-7-ylidene)methyl)-4,5-diphenylthieno[2,3-*c*]pyran-6-ium hexafluoroantimonate (3da)

Following the general procedure. 1-(Thiophen-2-yl)ethan-1-one (25.2 mg, 0.2 mmol), and diphenylacetylene (53.4 mg, 0.3 mmol) were used. The reaction was carried at 100 ℃ for 12 h. Purification via column chromatography on silica gel (petroleum ether /ethyl acetate = 1:1, v/v, then petroleum ether /dichloromethane /ethyl acetate = 1:1:4, v/v/v, then dichloromethane/methanol = 20:1, v/v) afforded **3da** as a dark purple solid (31.3 mg, 38% yield). ^{1}H NMR ($CDCl_3$, 400 MHz): δ (ppm) 8.13 (d, J = 2.8 Hz, 2H), 7.41-7.39 (m, 6H), 7.25-7.24 (m, 4H), 7.20-7.18 (m, 4H), 7.15 (d, J = 5.2 Hz, 2H), 7.05 (t, J = 7.6 Hz, 2H), 6.80 (t, J = 8.0 Hz, 4H), 6.28 (s, 1H). ^{13}C NMR ($CDCl_3$, 100 MHz): δ (ppm) 163.11, 155.72, 149.06, 140.84, 133.15, 130.52, 130.21, 130.05, 129.51, 129.41, 129.15, 128.74, 128.24, 126.13, 121.47, 110.14, 87.04. HRMS (ESI$^+$): calcd for $C_{39}H_{25}O_2S_2^+$, 589.1290; found 589.1287.

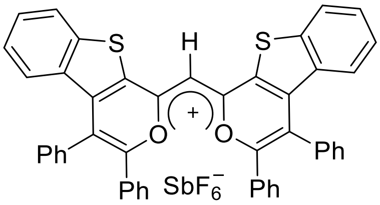

(*Z*)-1-((3,4-Diphenyl-1*H*-benzo[4,5]thieno[2,3-*c*]pyran-1-ylidene)methyl)-3,4-diphenylbenzo[4,5]thieno[2,3-*c*]pyran-2-ium hexafluoroantimonate (3ea)

Following the general procedure. 1-(Benzo[*b*]thiophen-2-yl)ethan-1-one (35.3 mg, 0.2 mmol), and diphenylacetylene (53.4 mg, 0.3 mmol) were used. The reaction was carried at 100 ℃ for 12 h. Purification via column chromatography on silica gel (petroleum ether /ethyl acetate = 1:1, v/v, then petroleum ether /dichloromethane /ethyl acetate = 1:1:4, v/v/v, then dichloromethane/methanol = 20:1, v/v) afforded **3ea** as a dark purple solid (50.9 mg, 55% yield). ^{1}H NMR ($CDCl_3$, 400 MHz): δ (ppm)

8.11 (d, J = 8.0 Hz, 2H), 7.65-7.61 (m, 2H), 7.54-7.48 (m, 6H), 7.38-7.36 (m, 4H), 7.24-7.22 (m, 4H), 7.20-7.18 (m, 2H), 7.08-7.04 (m, 2H), 6.84-6.80 (m, 4H), 6.73 (d, J = 8.4 Hz, 2H), 6.43 (s, 1H). ^{13}C NMR ($CDCl_3$, 100 MHz): δ (ppm) 162.53, 157.15, 144.36, 144.05, 134.20, 132.74, 131.28, 130.55, 130.33, 129.82, 129.77, 129.55, 129.47, 128.22, 127.25, 126.27, 123.97, 122.31, 89.30. HRMS (ESI^+): calcd for $C_{47}H_{29}O_2S_2^+$, 689.1603; found 689.1605.

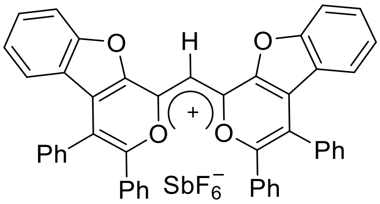

(*Z*)-1-((3,4-Diphenyl-1*H*-pyrano[3,4-*b*]benzofuran-1-ylidene)methyl)-3,4-dipheny lpyrano[3,4-*b*]benzofuran-2-ium hexafluoroantimonate (3fa)

Following the general procedure. 1-(Benzofuran-2-yl)ethan-1-one (32.0 mg, 0.2 mmol), and diphenylacetylene (53.4 mg, 0.3 mmol) were used. The reaction was carried at 100 °C for 12 h. Purification via column chromatography on silica gel (petroleum ether /ethyl acetate = 1:1, v/v, then petroleum ether /dichloromethane /ethyl acetate = 1:1:4, v/v/v, then dichloromethane/methanol = 20:1, v/v) afforded **3fa** as a dark purple solid (35.7 mg, 40% yield). ^{1}H NMR ($CDCl_3$, 400 MHz): δ (ppm) 7.83-7.79 (m, 2H), 7.72-7.68 (m, 2H), 7.52-7.47 (m, 6H), 7.42-7.39 (m, 4H), 7.34 (d, J = 7.6 Hz, 4H), 7.28-7.,24 (m, 2H), 7.11 (t, J = 7.6 Hz, 2H), 7.02 (s, 1H), 6.96 (d, J = 8.4 Hz, 2H), 6.87 (t, J = 8.0 Hz, 4H). HRMS (ESI^+): calcd for $C_{47}H_{29}O_4^+$, 657.2060; found 657.2056.

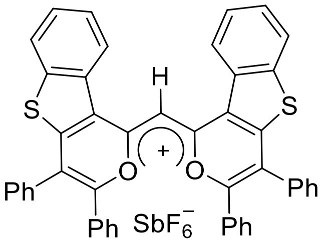

(*Z*)-1-((3,4-Diphenyl-1*H*-benzo[4,5]thieno[3,2-*c*]pyran-1-ylidene)methyl)-3,4-diph enylbenzo[4,5]thieno[3,2-*c*]pyran-2-ium hexafluoroantimonate (3ga)

Following the general procedure. 1-(Benzo[b]thiophen-3-yl)ethan-1-one (35.3 mg, 0.2 mmol), and diphenylacetylene (53.4 mg, 0.3 mmol) were used. The reaction was carried at 100 ℃ for 12 h. Purification via column chromatography on silica gel (petroleum ether /ethyl acetate = 1:1, v/v, then petroleum ether /dichloromethane /ethyl acetate = 1:1:4, v/v/v, then dichloromethane/methanol = 20:1, v/v) afforded **3ga** as a dark purple solid (54.6 mg, 59% yield). ^{1}H NMR (CD_3CN, 400 MHz): δ (ppm) 8.69 (d, *J* = 8.0 Hz, 2H), 8.15 (d, *J* = 8.4 Hz, 2H), 7.84 (t, *J* = 7.8 Hz, 2H), 7.74 (t, *J* = 7.6 Hz, 2H), 7.55-7.50 (m, 7H), 7.46-7.44 (m, 4H), 7.35-7.33 (m, 4H), 7.11(t, *J* = 7.2 Hz, 2H), 6.86 (t, *J* = 8.0 Hz, 4H). ^{13}C NMR ($CDCl_3$, 100 MHz): δ (ppm) 162.54, 159.82, 155.86, 139.86, 133.11, 133.01, 131.16, 129.80, 129.69, 129.60, 129.59, 129.38, 128.16, 128.14, 127.60, 123.81, 123.20, 122.35, 89.68. HRMS (ESI$^+$): calcd for $C_{47}H_{29}O_2S_2^+$, 689.1603; found 689.1605.

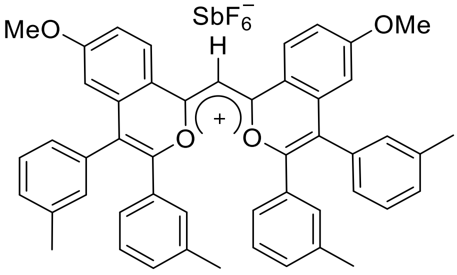

(*Z*)-6-Methoxy-1-((6-methoxy-3,4-di-*m*-tolyl-1*H*-isochromen-1-ylidene)methyl)-3,4-di-*m*-tolylisochromenylium hexafluoroantimonate (3ab)

Following the general procedure. 4-Methoxyacetophenone (30.0 mg, 0.2 mmol), and 1,2-di-*m*-tolylethyne (61.9 mg, 0.3 mmol) were used. The reaction was carried at 100 ℃ for 12 h. Purification via column chromatography on silica gel (petroleum ether /ethyl acetate = 1:1, v/v, then petroleum ether /dichloromethane /ethyl acetate = 1:1:4, v/v/v, then dichloromethane/methanol = 20:1, v/v) afforded **3ab** as a dark purple solid (69.7 mg, 75% yield). ^{1}H NMR ($CDCl_3$, 400 MHz): δ (ppm) 8.82 (d, *J* = 9.2 Hz, 2H), 7.56 (s, 1H), 7.51-7.48 (m, 2H), 7.30 (t, *J* = 7.6 Hz, 2H), 7.21 (d, *J* = 7.6 Hz, 2H), 7.00 (m, 4H), 6.92 (m, 2H), 6.85 (d, *J* = 7.6 Hz, 2H), 6.74 (d, *J* = 7.6 Hz, 2H), 6.70 (d, *J* = 2.8 Hz, 2H), 6.59 (t, *J* = 7.6 Hz, 2H), 3.87 (s, 6H), 2.33 (s, 6H), 1.84 (s, 6H). ^{13}C NMR ($CDCl_3$, 100 MHz): δ (ppm) 168.48, 166.96, 152.54, 139.48,

139.09, 137.61, 132.97, 131.43, 131.25, 130.77, 130.56, 129.73, 129.55, 129.17, 127.90, 127.55, 126.35, 121.77, 119.06, 116.41, 110.13, 109.45, 86.80, 56.26, 29.85, 21.56. HRMS (ESI$^+$): calcd for $C_{49}H_{41}O_4^+$, 693.2999; found 693.2997.

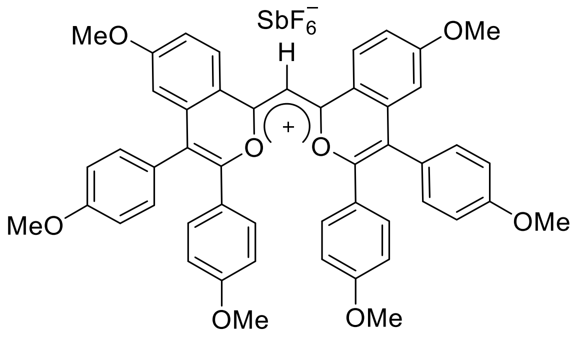

(*Z*)-6-Methoxy-1-((6-methoxy-3,4-bis(4-methoxyphenyl)-1*H*-isochromen-1-ylidene)methyl)-3,4-bis(4-methoxyphenyl)isochromenylium hexafluoroantimonate (3ac)

Following the general procedure. 4-Methoxyacetophenone (30.0 mg, 0.2 mmol), and 1,2- 1,2-bis(4-methoxyphenyl)ethyne (71.5 mg, 0.3 mmol) were used. The reaction was carried at 100 ℃ for 12 h. Purification via column chromatography on silica gel (petroleum ether /ethyl acetate = 1:1, v/v, then petroleum ether /dichloromethane /ethyl acetate = 1:1:4, v/v/v, then dichloromethane/methanol = 20:1, v/v) afforded **3ac** as a dark purple solid (49.5 mg, 50% yield). ^{1}H NMR (CD_3CN, 400 MHz): δ (ppm) 8.63 (d, J = 9.2 Hz, 2H), 7.36-7.33 (m, 3H), 7.20-7.16 (m, 4H), 7.11-7.08 (m, 4H), 7.03-6.99 (m, 4H), 6.70 (d, J = 2.4 Hz, 2H), 6.28-6.25 (m, 4H), 3.824 (s, 6H), 3.821 (s, 6H), 3.62 (s, 6H). ^{13}C NMR (CD_3CN, 100 MHz): δ (ppm) 169.10, 167.43, 161.31, 160.75, 154.28, 141.60, 132.98, 131.85, 131.02, 126.02, 124.31, 121.24, 119.96, 116.91, 115.51, 113.68, 108.31, 87.15, 56.85, 55.98, 55.57. HRMS (ESI$^+$): calcd for $C_{49}H_{41}O_8^+$, 757.2796; found 757.2797.

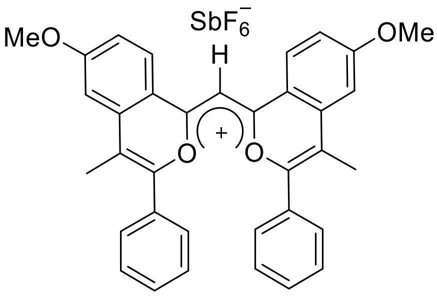

(*Z*)-6-Methoxy-1-((6-methoxy-4-methyl-3-phenyl-1*H*-isochromen-1-ylidene)methyl)-4-methyl-3-phenylisochromenylium hexafluoroantimonate (3ad)

Following the general procedure. 4-Methoxyacetophenone (30.0 mg, 0.2 mmol), and 1-phenyl-1-propyne (34.9 mg, 0.3 mmol) were used. The reaction was carried at 100 ℃ for 12 h. Purification via column chromatography on silica gel (petroleum ether /ethyl acetate = 1:1, v/v, then petroleum ether /dichloromethane /ethyl acetate = 1:1:4, v/v/v, then dichloromethane/methanol = 20:1, v/v) afforded **3ad** as a dark purple solid (26.2 mg, 35% yield). ^{1}H NMR (CD_3CN, 400 MHz): δ (ppm) 8.60 (d, J = 9.6 Hz, 2H), 7.40-7.39 (m, 2H), 7.39-7.37 (m, 3H), 7.34-7.29 (m, 4H), 7.26 (d, J = 2.4 Hz, 2H), 7.15-7.11 (m, 4H), 4.06 (s, 6H), 2.33 (s, 6H). HRMS (ESI$^+$): calcd for $C_{35}H_{29}O_4^+$, 513.2060; found 513.2059.

VII. Mechanistic study

Control experiments

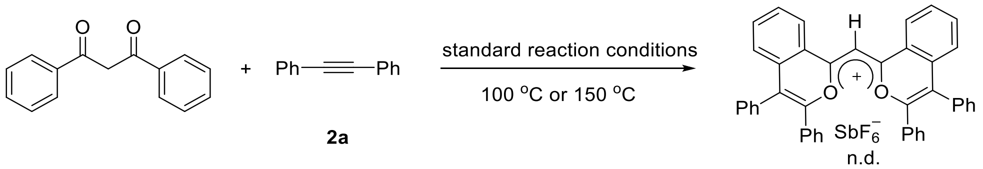

A Schlenk tube with a magnetic stir bar was charged with $[Cp^*RhCl_2]_2$ (0.005 mmol), $AgSbF_6$ (0.02 mmol), Ag_2CO_3 (0.3 mmol), $Cu(OAc)_2 \cdot H_2O$ (0.04 mmol), $NaSbF_6$ (0.15 mmol), 1,3-diphenylpropane-1,3-dione (0.2 mmol), alkyne (0.3 mmol), and DCE (0.5 mL) under an N_2 atmosphere. The resulting solution was stirred at room temperature for 10 min and then at 100 ℃ or 150 ℃ for 12 h. The flavylium fluorophore could not be detected both at 100 ℃ and 150 ℃.

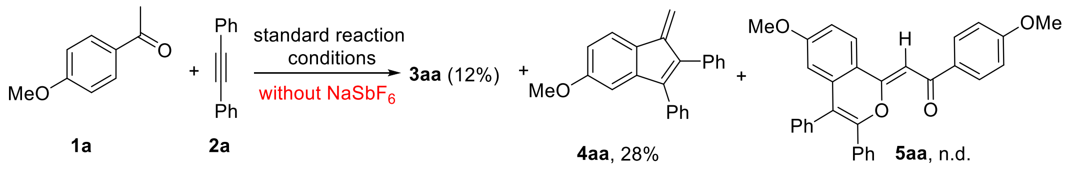

A Schlenk tube with a magnetic stir bar was charged with [Cp*RhCl$_2$]$_2$ (0.005 mmol), AgSbF$_6$ (0.02 mmol), Ag$_2$CO$_3$ (0.3 mmol), Cu(OAc)$_2$·H$_2$O (0.04 mmol), 4-methoxyacetophenone (0.2 mmol), diphenylacetylene (0.3 mmol), and DCE (0.5 mL) under an N$_2$ atmosphere. The resulting solution was stirred at room temperature for 10 min and then at 100 ℃ for 12 h. The resulting solution was cooled to ambient temperature, and diluted with 10 mL of dichloromethane. The obtained organic extracts was evaporated under reduced pressure and the residue was absorbed into small amounts of silica gel. Purification was performed by column chromatography on silica gel to provide product **3aa** in 12% yield and **4aa** in 28% yield. 5-methoxy-1-methylene-2,3-diphenyl-1*H*-indene (**4aa**): ^{1}H NMR (CDCl$_3$, 400 MHz): δ (ppm) 7.63 (d, J = 8.0 Hz, 1H), 7.34-7.27 (m, 7H), 7.23-7.18 (m, 3H), 6.91 (d, J = 2.0 Hz, 1H), 6.81 (dd, J_1 = 8.0 Hz, J_2 = 2.4 Hz, 1H), 6.13 (s, 1H), 5.63 (s, 1H), 3.83 (s, 3H). ^{13}C NMR (CDCl$_3$, 100 MHz): δ (ppm) 160.61, 147.15, 144.51, 141.47, 138.88, 134.75, 134.60, 130.82, 129.55, 129.12, 128.42, 128.11, 127.55, 127.08, 120.84, 112.88, 110.83, 106.57, 55.75.

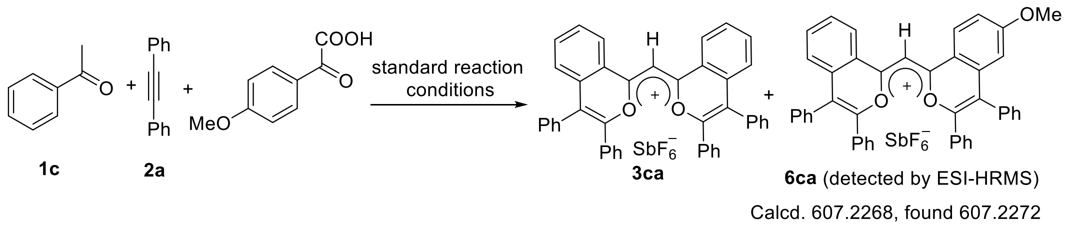

A Schlenk tube with a magnetic stir bar was charged with [Cp*RhCl$_2$]$_2$ (0.005 mmol), AgSbF$_6$ (0.02 mmol), Ag$_2$CO$_3$ (0.3 mmol), Cu(OAc)$_2$·H$_2$O (0.04 mmol), NaSbF$_6$ (0.15 mmol), acetophenone (0.1 mmol), 2-(4-methoxyphenyl)-2-oxoacetic acid (0.1 mmol), diphenylacetylene (0.3 mmol), and DCE (0.5 mL) under an N$_2$ atmosphere. The resulting solution was stirred at room temperature for 10 min and then at 100 ℃ for 12 h. The reaction mixture was used for high resolution

electrospray ionization mass spectrometry (ESI-HRMS) detection (Supplementary Figure 3).

Radical trapping experiments

A Schlenk tube with a magnetic stir bar was charged with $[Cp^*RhCl_2]_2$ (0.005 mmol), $AgSbF_6$ (0.02 mmol), Ag_2CO_3 (0.3 mmol), $Cu(OAc)_2 \cdot H_2O$ (0.04 mmol), $NaSbF_6$ (0.15 mmol), 4-methoxyacetophenone (0.2 mmol), diphenylacetylene (0.3 mmol), TEMPO and DCE (0.5 mL) under an N_2 atmosphere. The resulting solution was stirred at room temperature for 10 min and then at 100 ℃ for 12 h. The resulting solution was cooled to ambient temperature, and diluted with 10 mL of dichloromethane. The obtained organic extracts was evaporated under reduced pressure and the residue was absorbed into small amounts of silica gel. Purification was performed by column chromatography on silica gel to provide product **3aa** (Supplementary Table 2).

A Schlenk tube with a magnetic stir bar was charged with $[Cp^*RhCl_2]_2$ (0.005 mmol), $AgSbF_6$ (0.02 mmol), Ag_2CO_3 (0.3 mmol), $Cu(OAc)_2 \cdot H_2O$ (0.04 mmol), $NaSbF_6$ (0.15 mmol), 2-acetylbenzo[*b*]thiophene (0.2 mmol), diphenylacetylene (0.3 mmol), TEMPO (0.2 mmol) and DCE (0.5 mL) under an N_2 atmosphere. The resulting solution was stirred at room temperature for 10 min and then at 100 ℃ for 12 h. The resulting solution was cooled to ambient temperature, and diluted with 10 mL of dichloromethane. The obtained organic extracts was evaporated under reduced pressure and the residue was absorbed into small amounts of silica gel. Purification was performed by column chromatography on silica gel to provide product **7ea** in 39% yield (Supplementary Figure 4). Only trace amount of product **7ea** could be detected when the reaction was conducted without the addition of TEMPO. 3,4-Diphenyl-1*H*-benzo[4,5]thieno[2,3-*c*]pyran-1-one (**7ea**): ^1H NMR ($CDCl_3$, 400 MHz): δ (ppm) 7.93 (td, J_1 = 8.4 Hz, J_2 = 0.8 Hz, 1H), 7.53-7.44 (m, 4H), 7.40-7.36 (m, 4H), 7.26-7.19 (m, 3H), 7.13-7.09 (m, 1H), 6.67 (d, J = 8.4 Hz, 1H). ^{13}C NMR ($CDCl_3$, 100 MHz): δ (ppm) 159.12, 154.12, 143.95, 143.42, 135.16, 134.43, 132.61, 131.24, 129.43, 129.41, 129.24, 128.96, 128.39, 128.06, 126.31, 124.96, 123.67,

123.58, 116.73. HRMS (ESI^+): calcd for $[C_{23}H_{14}O_2S + H]^+$, 355.0787; found 355.0790.

A Schlenk tube with a magnetic stir bar was charged with $AgSbF_6$ (0.02 mmol), Ag_2CO_3 (0.3 mmol), $Cu(OAc)_2 \cdot H_2O$ (0.04 mmol), $NaSbF_6$ (0.15 mmol), 2-acetylbenzo[*b*]thiophene (0.2 mmol), diphenylacetylene (0.3 mmol), TEMPO (0.2 mmol) and DCE (0.5 mL) under an N_2 atmosphere. The resulting solution was stirred at room temperature for 10 min and then at 100 ℃ for 12 h. The resulting solution was cooled to ambient temperature, and diluted with 10 mL of dichloromethane. The obtained organic extracts was evaporated under reduced pressure and the residue was absorbed into small amounts of silica gel. Purification was performed by column chromatography on silica gel to provide product **8ea** in 18% yield (Supplementary Figure 5). 1,2-bis(Benzo[*b*]thiophen-2-yl)ethane-1,2-dione (**8ea**): ^{1}H NMR ($CDCl_3$, 400 MHz): δ (ppm) 8.33 (s, 2H), 7.95-7.93 (m, 4H), 7.54 (t, J = 7.6 Hz, 2H), 7.44 (t, J = 7.6 Hz, 2H). ^{13}C NMR ($CDCl_3$, 100 MHz): δ (ppm) 184.45, 143.96, 139.18, 138.90, 135.60, 128.82, 127.05, 125.56, 123.17.

Electron paramagnetic resonance (EPR) experiments

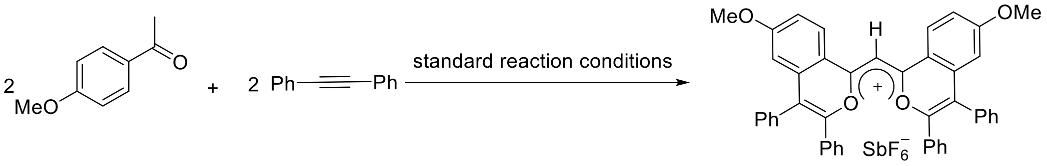

1a 2a 3aa

A Schlenk tube with a magnetic stir bar was charged with $[Cp^*RhCl_2]_2$ (0.005 mmol), $AgSbF_6$ (0.02 mmol), Ag_2CO_3 (0.3 mmol), $Cu(OAc)_2 \cdot H_2O$ (0.04 mmol), $NaSbF_6$ (0.15 mmol), 4-methoxyacetophenone (0.2 mmol), diphenylacetylene (0.3 mmol) and DCE (0.5 mL) under an N_2 atmosphere. The resulting solution was stirred at room temperature for 10 min and then at 100 ℃ for 1.5 h. The resulting mixture was used for EPR measurement (Supplementary Figure 6a): modulation frequency, 100.00 kHz; modulation amplitude, 2.00 G; sweep width, 80.00 G; time constant, 40.96 ms; conversion time, 80.00 ms; sweep time, 81.92 ms; receiver gain, 1.00×10^3. The microwave frequency, 9.435336 GHz; power, 18.92 mW.

A Schlenk tube with a magnetic stir bar was charged with $[Cp^*RhCl_2]_2$ (0.005 mmol), $AgSbF_6$ (0.02 mmol), Ag_2CO_3 (0.3 mmol), $Cu(OAc)_2 \cdot H_2O$ (0.04 mmol), $NaSbF_6$ (0.15 mmol) and DCE (0.5 mL) under an N_2 atmosphere. The resulting solution was stirred at room temperature for 10 min and then at 100 ℃ for 1.5 h. The resulting mixture was used for EPR measurement (Supplementary Figure 6b): modulation frequency, 100.00 kHz; modulation amplitude, 2.00 G; sweep width, 80.00 G; time constant, 40.96 ms; conversion time, 80.00 ms; sweep time, 81.92 ms; receiver gain, 1.00×10^3. The microwave frequency, 9.435336 GHz; power, 18.92 mW.

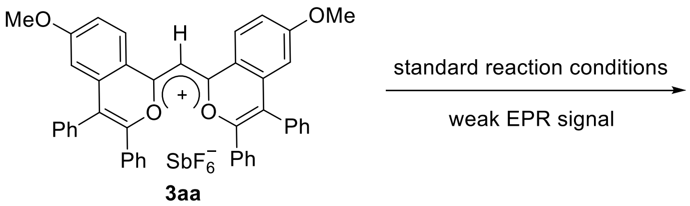

A Schlenk tube with a magnetic stir bar was charged with $[Cp^*RhCl_2]_2$ (0.005 mmol), $AgSbF_6$ (0.02 mmol), Ag_2CO_3 (0.3 mmol), $Cu(OAc)_2 \cdot H_2O$ (0.04 mmol), $NaSbF_6$ (0.15 mmol), **3aa** (0.1 mmol) and DCE (0.5 mL) under an N_2 atmosphere. The resulting solution was stirred at room temperature for 10 min and then at 100 ℃ for 1.5 h. The resulting mixture was used for EPR measurement (Supplementary Figure 6c): modulation frequency, 100.00 kHz; modulation amplitude, 2.00 G; sweep width, 80.00 G; time constant, 40.96 ms; conversion time, 80.00 ms; sweep time, 81.92 ms; receiver gain, 1.00×10^3. The microwave frequency, 9.435336 GHz; power, 18.92 mW.

VIII. Photophysical properties of the representative products

The photophysical properties of the BFFs were measured in chromatographically pure CH_2Cl_2 at 40.0 μM. UV/Vis spectra experiments were conducted on a HITACHI U-2910. Fluorescence spectra and absolute quantum yields were measured on a Horiba Fluorolog-3 fluorescence spectrometer with a calibrated integrating sphere system. The peaks of the water Raman spectrum and the F-1031 lamp spectrum are at

396 nm and 467 nm, respectively. The PMT dark counts are 168 cps (Approx.). The results of signal to noise computation show that S: R is 66674.32991, higher than the 1st standard deviation (24363.75749). Fluorescence spectra were measured upon the excitation wavelength at 563 nm, 562 nm, 570 nm, 554 nm, 597 nm, 605 nm, 566 nm, 547 nm, 565 nm, 533 nm for **3aa**, **3ba**, **3ca**, **3da**, **3ea**, **3fa**, **3ga**, **3ab**, **3ac**, **3ad**, respectively. Excitation spectra were detected upon the maximum emission wavelength.

IX. DFT calculation[4, 5, 6]

Computational Methods

The DFT calculations were carried out with the GAUSSIAN 09 series of programs. Density functional theory B3LYP1 with a standard 6-31+G(d) basis set was used for geometry optimizations. Harmonic frequency calculations were performed for stationary point to confirm them as a local minima.

Supplementary References

1. Kang, K. W., Moseley, K. & Maitlis, P. M. Pentamethylcyclopentadienylrhodium and -iridium halides. I. Synthesis and properties. *J. Am. Chem. Soc.* **91**, 5970–5977 (1969).
2. Hyder, Z., Ruan, J. & Xiao, J. Hydrogen-bond-directed catalysis: faster, regioselective and cleaner Heck arylation of electron-rich olefins in alcohols. *Chem. Eur. J.* **14**, 5555–5566 (2008).
3. Park, K., Bae, G., Moon, J., Choe, J., Song, K. H. & Lee, S. Synthesis of symmetrical and unsymmetrical diarylalkynes from propiolic acid using palladium-catalyzed decarboxylative coupling. *J. Org. Chem.* **75**, 6244–6251 (2010).
4. Becke, A. D. Density-functional thermochemistry. III. The role of exact exchange. *J. Chem. Phys.* **98**, 5648–5652 (1993).
5. Lee, C., Yang, W. & Parr, R. G. Development of the colle-salvetti correlation-energy formula into a functional of the electron density. *Phys. Rev. B*

37, 785 (1988).

6. Gaussian 09, Revision D.01, Frisch, M. J., Trucks, G. W., Schlegel, H. B., Scuseria, G. E., Robb, M. A., Cheeseman, J. R., Scalmani, G., Barone, V., Mennucci, B., Petersson, G. A., Nakatsuji, H., Caricato, M., Li, X., Hratchian, H. P., Izmaylov, A. F., Bloino, J., Zheng, G., Sonnenberg, J. L., Hada, M., Ehara, M., Toyota, K., Fukuda, R., Hasegawa, J., Ishida, M., Nakajima, T., Honda, Y., Kitao, O., Nakai, H., Vreven, T., Montgomery, J. A., Jr., Peralta, J. E., Ogliaro, F., Bearpark, M., Heyd, J. J., Brothers, E., Kudin, K. N., Staroverov, V. N., Kobayashi, R., Normand, J., Raghavachari, K., Rendell, A., Burant, J. C., Iyengar, S. S., Tomasi, J., Cossi, M., Rega, N., Millam, N. J., Klene, M., Knox, J. E., Cross, J. Bakken, B., V., Adamo, C., Jaramillo, J., Gomperts, R., Stratmann, R. E., Yazyev, O., Austin, A. J., Cammi, R., Pomelli, C., Ochterski, J. W., Martin, R. L., Morokuma, K., Zakrzewski, V. G., Voth, G. A., Salvador, P., Dannenberg, J. J., Dapprich, S., Daniels, A. D., Farkas, Ö., Foresman, J. B., Ortiz, J. V., Cioslowski, J., Fox, D. J., Gaussian, Inc., Wallingford CT, **2013**.
