# Supplementary material for: Acyl radical to rhodacycle addition and cyclization relay to access butterfly flavylium fluorophores
Source: Nat Commun. 2019 Dec 11;10:5664. doi: 10.1038/s41467-019-13611-6 (PMC6906420; doi:10.1038/s41467-019-13611-6)
Supplement: Supplementary file 2 — Description of Additional Supplementary Files [file 41467_2019_13611_MOESM2_ESM.pdf]

<u>Description of Additional Supplementary Files</u>

File Name: Supplementary Data 1

Description: The DFT calculation details (cartesian coordinates) of product 3aa.
