# Supplementary material for: Acyl radical to rhodacycle addition and cyclization relay to access butterfly flavylium fluorophores
Source: Nat Commun. 2019 Dec 11;10:5664. doi: 10.1038/s41467-019-13611-6 (PMC6906420; doi:10.1038/s41467-019-13611-6)
Supplement: Supplementary file 3 — Supplementary Data 1 [file 41467_2019_13611_MOESM3_ESM.pdf]

B3LYP Geometries for the Optimized Compounds and Transition State (3aa)

C	-2.50728000	3.57003900	0.57767700
H	-1.57903100	4.11736000	0.69377700
C	-3.68972100	4.24900400	0.74154500
H	-3.70747100	5.30944600	0.97079300
H	1.57925700	4.11700600	-0.69554100
C	3.72976200	1.48160800	-0.19396100
H	3.70778500	5.30912100	-0.97201600
C	4.92399600	3.56131200	-0.61095400
C	-1.26934500	1.45884000	0.05649600
O	1.34309000	0.12999300	0.14819500
C	4.93675800	2.19275200	-0.34597800
C	3.72457800	0.05223400	0.07496500
O	-1.34308900	0.13012100	-0.14919400
C	-2.48879100	2.18025200	0.28123900
C	2.52254200	-0.57872300	0.22364500
H	5.87187900	1.65788600	-0.25364500
C	5.01750600	-0.68298100	0.23389300
C	7.31895400	3.72847200	-0.65037800
C	-2.52254700	-0.57862400	-0.22419000
C	-3.72962200	1.48171400	0.19383200
C	2.26076900	-2.00055700	0.52807500

C	5.83600000	-0.93894300	-0.87880500
C	5.44266900	-1.10567600	1.50233400
H	7.46212900	3.30082800	0.34828200
H	7.46353900	2.95791900	-1.41596500
C	-3.72452500	0.05229600	-0.07488900
C	-2.26084300	-2.00040900	-0.52896200
C	-4.93658200	2.19283800	0.34622600
C	1.29049700	-2.33236900	1.49177100
C	2.96324600	-3.03299900	-0.11692300
H	5.51638900	-0.61660400	-1.86740200
C	7.04891100	-1.61569800	-0.72688000
C	6.65743600	-1.77965000	1.65418700
H	4.81821500	-0.91098600	2.37041800
C	-5.01755100	-0.68293200	-0.23292700
C	-2.96306800	-3.03302300	0.11603500
C	-1.29086900	-2.33196900	-1.49304700
C	-4.92377000	3.56147300	0.61082700
H	-5.87171900	1.65790000	0.25446200
H	0.73476800	-1.54360100	1.98940900
C	1.05223000	-3.66544900	1.82342600
C	2.71191300	-4.36591000	0.20945800
H	3.70129900	-2.79668000	-0.87526900

H	7.66768200	-1.81545100	-1.59773000
C	7.46202500	-2.03829000	0.54085400
C	-5.44382700	-1.10535100	-1.50108200
C	-5.83508500	-0.93910600	0.88043800
H	-3.70086300	-2.79690000	0.87469300
C	-2.71178600	-4.36584900	-0.21072100
C	2.50749600	3.56978800	-0.57885400
H	0.00014300	3.12764300	-0.00036000
C	2.48894600	2.18008400	-0.28202900
C	3.68997500	4.24875300	-0.74243100
C	0.00007200	2.04962300	-0.00046200
C	1.26943200	1.45872600	-0.05743100
C	-1.05265100	-3.66496900	-1.82507100
H	-0.73534400	-1.54307200	-1.99071200
O	-6.02076400	4.32344700	0.76728000
H	0.31102500	-3.90722000	2.58004100
C	1.76333100	-4.68611000	1.18500300
H	3.25995800	-5.15431900	-0.29909800
H	8.40450300	-2.56569300	0.65953700
C	-6.65874400	-1.77928300	-1.65201200
C	-7.04814200	-1.61580000	0.72942900
H	-5.51461600	-0.61695700	1.86882100

H	-3.25963000	-5.15439700	0.29783500
C	-1.76350200	-4.68579600	-1.18664100
H	-0.31167500	-3.90654100	-2.58197500
H	1.57672700	-5.72480100	1.44381000
H	-6.97483100	-2.10360700	-2.63977100
C	-7.46236400	-2.03812600	-0.53803100
H	-7.66616100	-1.81573000	1.60077100
H	-1.57693800	-5.72442300	-1.44573500
H	-7.46269400	2.95806600	1.41736500
H	-8.40498200	-2.56544000	-0.65599000
O	6.02102400	4.32329200	-0.76716600
H	8.02534000	4.54318600	-0.80847300
H	-4.82014100	-0.91048000	-2.36967400
C	-7.31871300	3.72847100	0.65152100
H	-8.02507300	4.54314700	0.80992500
H	-7.46252900	3.30060500	-0.34695100
H	6.97266900	-2.10417200	2.64215100
